# Stem cell-based approaches for developmental neurotoxicity testing

**DOI:** 10.3389/ftox.2024.1402630

**Published:** 2024-08-22

**Authors:** Joy Ku, Prashanth Asuri

**Affiliations:** Department of Bioengineering, Santa Clara University, Santa Clara, CA, United States

**Keywords:** developmental neurotoxicity (DNT), predictive modeling, integrated approaches for testing and assessment (IATA), new approach methodologies (NAM), neural stem cell (NSC)

## Abstract

Neurotoxicants are substances that can lead to adverse structural or functional effects on the nervous system. These can be chemical, biological, or physical agents that can cross the blood brain barrier to damage neurons or interfere with complex interactions between the nervous system and other organs. With concerns regarding social policy, public health, and medicine, there is a need to ensure rigorous testing for neurotoxicity. While the most common neurotoxicity tests involve using animal models, a shift towards stem cell-based platforms can potentially provide a more biologically accurate alternative in both clinical and pharmaceutical research. With this in mind, the objective of this article is to review both current technologies and recent advancements in evaluating neurotoxicants using stem cell-based approaches, with an emphasis on developmental neurotoxicants (DNTs) as these have the most potential to lead to irreversible critical damage on brain function. In the next section, attempts to develop novel predictive model approaches for the study of both neural cell fate and developmental neurotoxicity are discussed. Finally, this article concludes with a discussion of the future use of *in silico* methods within developmental neurotoxicity testing, and the role of regulatory bodies in promoting advancements within the space.

## 1 Introduction

### 1.1 Manuscript overview

Developmental toxicity is defined as the effects that interfere with normal development before or after birth, resulting from either pre- or postnatal exposure. These effects can manifest at any time point, with major manifestations including functional deficits, altered growth, or structural abnormalities, according to the OECD Guidance Document 43. GD 43 was published as part of a series on testing and assessments for human health, which aimed to provide additional guidance on the overall testing approaches used for DNTs (Grandjean and Landrigan, 2006; OECD, 2008). It currently supplements testing guidelines published by the United States Environmental Protection Agency (USEPA) and the Organization for Economic Cooperation and Development (OECD). While a broad range of toxins and toxicants can be considered DNTs, this paper will predominantly refer to DNTs as chemical and small-molecules that have developmental neurotoxic effects.

Guidelines set by the USEPA and OECD for DNT testing mainly focus on *in vivo* testing – specifically in murine models – to assess motor and autonomic function, convulsive behaviors, and other “unusual or abnormal behaviors” (USEPA United States Environmental Protection Agency, 1998; OECD, 2007; Makris et al., 2009). In addition, the OECD Testing Guideline (TG) 443 specifically focuses on assessing the potential impact of pre- and post-natal chemical exposure on both the developing nervous and immune systems (OECD, 2018). While these guidelines represent the best assessment for DNT human health risk, TG 443 requires extensive amounts of time, animal, and financial resources to conduct. The highly involved nature of *in vivo* testing, alongside inadequate regulations and policies, potentially explains the lack of testing among the roughly 87,000 commercially used chemical substances (TSCA Chemical Substance Inventory, 2023). To date, approximately only 165 chemicals have been assessed using either the EPA or OECD TG 443 guidelines (Crofton and Mundy, 2021). Animal-based test methods are also limited in their interpretation due to issues with variability, precision, and uncertain human relevance (OECD work on *in vitro*). Recognizing the drawbacks of *in vivo* testing, efforts are focused on developing alternative models for the evaluation of potential DNTs. Most recently, the OECD released a report on the use of non-animal testing methods for developmental neurotoxicity and has published a guidance document on evaluating data from *in vitro* testing batteries on DNTs (OECD, 2017; OECD, 2022; OECD, 2023). The USEPA is also developing a work plan to reduce vertebrate animal testing in their New Approach Methodologies (NAMs) (USEPA, 2021).

It is recognized that many of the models and *in vitro* studies described below may refer more broadly to general neuronal or other toxicity. Echoing the DNT testing guidelines set forth by the OECD, which incorporates established assays originally developed for other investigations of toxicity, these examples are meant to serve as points of discussion in which applications of such methodologies can be expanded to cover applications within DNT testing. In other words, this paper builds on existing literature with a focus on current strategies for stem cell-based approaches to investigating potential DNTs, before further expanding on the novel use of predictive models in this space. Predictive models can be a powerful tool in toxicity testing, especially when used for initial screenings of chemicals. By utilizing a predictive model to isolate potential DNTs, the experimental space can be reduced which allows for more effective use of testing resources.

### 1.2 Developmental neurotoxicants – Definition, classification, and implications

The first neurotoxicants established to also be DNTs were identified in 2006 and included lead, methylmercury, arsenic, polychlorinated biphenyls (PCBs), and toluene (Grandjean and Landrigan, 2006). Since then, there have been several more identified: manganese, fluoride, perchloroethylene, organochlorine compounds such as dichlorodiphenyltrichloroethane (DDT), chlorpyrifos, and the polybrominated diphenyl ethers (Grandjean and Landrigan, 2014). Numerous other studies are currently investigating other neurotoxic compounds that may potentially affect brain development.

Developmental neurotoxicants (DNTs) may work specifically in preventing neural stem cell differentiation, by causing permanent changes in gene expression, damaging DNA, modifying key signaling proteins in essential pathways, among many others (Song et al., 2011). This impact on neural development can be distinct from the effect of stem cell toxicants, which are those that are cytotoxic to stem cells, and neurotoxicants, which are either cytotoxic or functional toxicants to neurons or glial cells. At the same time, there can be a significant amount of overlap between the three types of toxicants, as an identified DNT could potentially also be a functional toxicant to neurons after development. The lack of standard *in vitro* DNT testing methods remains an issue, with many teams relying on looking at endpoints which sometimes correspond with outcomes similar to stem cell toxicant or neurotoxicant activity, as will be discussed later. Therefore, working towards improving testing could allow for stricter distinctions between DNTs, stem cell toxicants, and neurotoxicants in the future. For these reasons, this paper includes established developmental neurotoxicants along with neurotoxicants that impact stem cell differentiation or other critical developmental processes (e.g., synaptogenesis) as DNTs.

The developing brain is highly susceptible to DNTs for several reasons, as DNTs cause adverse effects by interfering with the cascade of developmental processes. Neurogenesis occurs throughout different parts of the brain at various stages, and the introduction of harmful compounds at any time point can lead to the degeneration of neurons and synapses crucial for brain function (Abbott and Nigussie, 2021). Gliogenesis, the process in which glial cells such as astrocytes and oligodendrocytes rapidly proliferate, is also susceptible to the toxic effects of certain chemicals (Aschner et al., 1999). The blood brain barrier (BBB) is important for regulatory functions including nutrient metabolism and acts as a selective barrier, protecting the brain from certain potentially harmful compounds (Pardridge, 1983; Saili et al., 2017). The BBB is formed via angiogenesis which begins at around 8 weeks of gestation and peaks at roughly 35 weeks in humans (Mito et al., 1991; Marín-Padilla, 2012). Zhao et al. demonstrated that disruption of the BBB development during pregnancy by inflammatory agents can lead to long-lasting BBB dysfunction in offspring, potentially contributing to neuropsychiatric disorders (Zhao et al., 2022).

In one review paper, Heyer and Meredith state that the discussion of non-genetic environmental toxicants potentially affecting neural development has been mainly focused on their correlation with the onset of neurodevelopmental disorders (NDDs) such as autism, Attention Deficit/Hyperactivity Disorder (ADHD), and schizophrenia (Heyer and Meredith, 2017). The team goes on to detail the four most common pathophysiological mechanisms of DNTs in these NDDs: oxidative stress, immune system dysregulation, altered neurotransmitter systems (hyperserotonemia, dopamine dysfunction, neuronal excitability), and thyroid hormone disruptions. Using both human and animal findings, the team was also able to map sensitive time windows during brain development where exposure to specific DNTs and other chemical toxicants would increase the risk of certain NDDs.

The high risk of crucial developmental event disruptions due to prenatal exposure to DNTs cannot be overstated, emphasizing the importance of investigation and regulation of testing in this space. However, it is evident that current standards for toxicity testing may not be rigorous enough not only for screening potential DNTs, but perhaps neurotoxicants as a whole. To expand on this, the list of industrial chemicals that have a documented neurotoxic effect on adults range across numerous categories, including metals and inorganic compounds, organic solvents, organic substances, and pesticides. However, less than 20% of the most extensively regulated classes of these industrial chemicals have any information available for a thorough health-hazard assessment (National Research Council US). Even fewer of these compounds have been tested for selective toxicity in children, speaking to the lack of adequate regulations in place to identify DNTs. The magnitude of this issue could also be associated with the rising prevalence of developmental disabilities. From 1997 to 2017, there has been a 38.3% increase in the number of children in the US who were affected by developmental disabilities (Boyle et al., 2011; Zablotsky et al., 2019). Many of these disabilities are related to neurological development, some of which are the result of prolonged exposure of the brain to toxic substances. These agents can result in neurological disorders like epilepsy, schizophrenia, and dyslexia (Rice and Barone, 2000). The prevalence of potential DNTs in the environment, along with the detrimental consequences DNTs have on individuals and communities means it is imperative to find ways to effectively regulate testing, while also raising awareness and increasing understanding of developmental neurotoxicity.

### 1.3 Current methods to evaluate neurotoxicants

As previously discussed, efforts to move towards alternatives to *in vivo* testing for neurotoxicity have led to the development of *in vitro* testing batteries. The term *in vitro* in this context refers to the use of stem cells, stem cell derived neurons, or cultured neurons to analyze the specific impact of toxicants on cell fate (Sisnaiske et al., 2014; Kang et al., 2017; Mansel et al., 2019; Bu et al., 2020). Existing methods used for this purpose include assays studying cytotoxicity and cell viability, functionality, gene and protein expression, and cell morphology. These methods mostly involve two-dimensional (2D) cell cultures grown in monolayers, either in flat petri dishes or culture flasks, where cells attach to a cell-adherent surface such as plastic. As such, the applications for the following assays will be described using the conventional 2D cell culture method.

#### 1.3.1 Cytotoxicity and cell viability assays

**FIGURE 1:**
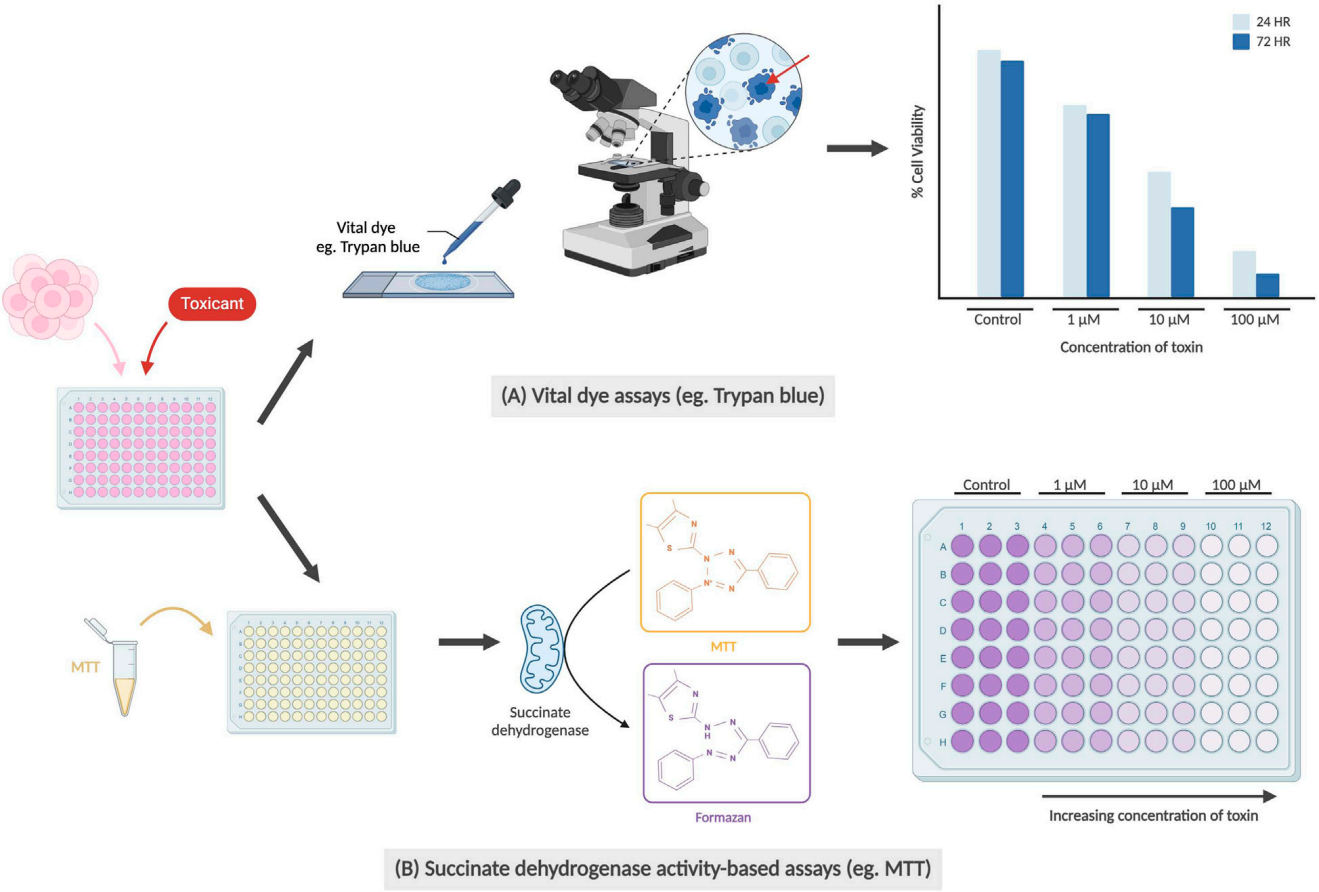
Examples of cell viability/cytotoxicity assays. **(A)** Vital dye assays are conducted by staining dead cells using dyes (e.g., Trypan blue) from which the percentage of viable cells (unstained) can be calculated. Red arrow indicates trypan blue staining of a non-viable cell with perforated membrane. The graph is not based on real data but is meant to represent conclusions drawn by previous teams. **(B)** Succinate dehydrogenase activity-based assays work by the conversion of water-soluble yellow MTT to purple formazan crystals by succinate dehydrogenase in the mitochondria of healthy cells. Measured absorbance of purple solution using a spectrophotometer is directly correlated with cell viability.

Cytotoxicity and cell viability assays are some of the most popular assays used in toxicology studies. They can be used to monitor cell health by detecting cell death or non-viability through assessing several features, with the most common being membrane integrity and metabolic activity (Figure 1). Assays detecting membrane integrity typically utilize “vital dyes,” such as trypan blue or propidium iodide, which selectively penetrate dead cells through their damaged membranes. Viable cells with intact membranes remain clear, allowing researchers to use a hemocytometer to count a small fraction of the cell suspension and extrapolating to find an estimated number of dead cells (Strober, 2015; Riss et al., 2019).

Succinate dehydrogenase activity-based assays (e.g., MTT, WST, or MTS), are commonly used to detect metabolic activity. These assays measure cell viability and proliferation by enzymatically reacting with succinate dehydrogenase in the mitochondria, with mitochondrial respiration catalyzing the reduction of the dye, such as MTT, into insoluble purple formazan crystals (Ghasemi et al., 2021). The number of viable cells can then be determined through colorimetric analysis after the cells have been lysed and processed. Both succinate dehydrogenase activity-based assays and dye exclusion assays allow researchers to efficiently determine the dose- and time-dependent cytotoxic effect of a drug or compound of interest.

Several teams have explored the use of human induced pluripotent stem cells (hiPSC) and hiPSC-derived neural stem cells (NSCs) for neurotoxicity testing, using cell viability assays as the main methodology for examining cytotoxicity (Valdiglesias et al., 2015; Pei et al., 2016; Kamata et al., 2020). NSCs proliferate and differentiate into numerous types of neural progenitor cells (NPCs) including neuronal and glial progenitors. These NPCs then proliferate, migrate, and differentiate into the three major cell types in the central nervous system (CNS) in the following temporally defined sequence: neurons appear first, followed by astrocytes, and finally oligodendrocytes (Okano and Temple, 2009; Zhao and Moore, 2018). Using mitochondrial MTS and cellular ATP assays, Kamata’s team from Japan found that compared to immortal cell lines Cos-7 and HepG2, hIPSCs and NPCs were more vulnerable to a majority of the 35 DNTs studied (as indicated by diminished IC_50_ values) (Kamata et al., 2020).

#### 1.3.2 Functional assays

**FIGURE 2:**
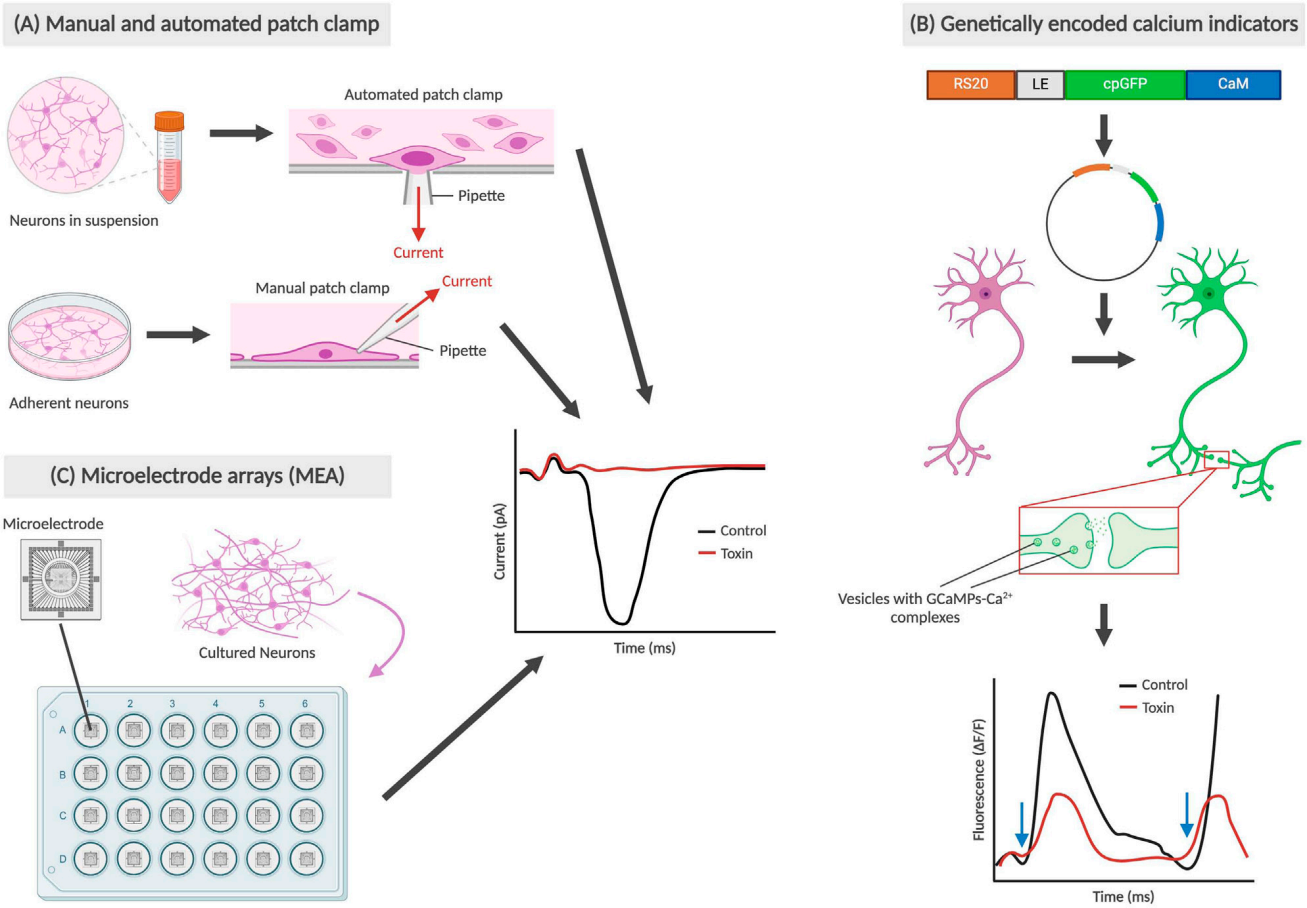
Types of functional assays for neural cells. The graphs are not based on real data; rather they are abstract representations that retain the same conclusions as described in prior studies. **(A)** Manual and automated patch clamp electrophysiology utilized adherent and suspended cell cultures respectively, with the electrical current generated during depolarization being recorded. **(B)** Genetically encoded calcium indicators (GECIs), such as GCaMPf6, bind with Ca^2+^ and allow for the observation of changes in fluorescence during synaptic transmissions (Zhang et al., 2023). Blue arrows indicate time points when cells are stimulated, and depolarization occurs. **(C)** Microelectrode arrays (MEA) also use cultured neurons on a special type of plate with microelectrodes embedded in the base, also producing a graph recording current during depolarization.

Functional assays, as depicted in Figure 2, measure a neuron’s electrophysiology and neurotransmission ability. In some cases, toxicity in neurons may manifest in non-obvious changes, such as changes in membrane potential or action potential propagation, in comparison to directly observable morphological changes like membrane integrity. The patch clamp technique is an electrophysiological method that directly measures membrane potential or current going across the cell membrane, and is currently the gold standard of functional assays (Hill and Stephens, 2021). This can be performed on single neurons, brain slices or live brains in sedated animals. In single cell applications, researchers place a glass micropipette electrode directly on a small area of the cell membrane and use suction to firmly seal the tip of the pipette to the cell. The tight seal provides extremely high resistance which allows for the detection of small voltage changes during action potential firing, while blocking external currents from surrounding cells (Covey and Carter, 2015). More recently, the automated patch clamp (APC) has become an increasingly popular application that allows for high-throughput screening using the same technique – with each APC chip commonly having either 384 or 768 wells (Bell and Dallas, 2018; Toh et al., 2020). Instead of manually attaching the pipette onto a single adherent cell, cell suspensions are applied to planar recording chips of varying substrate materials, including borosilicate glass, silicon, and polymer. Due to a combination of gravity and negative pressure, a high electrical resistance seal is formed between the chip and the immobilized cell which then allows for accurate recordings of membrane potential or current (Bell and Dallas, 2018; Obergrussberger et al., 2022).

In one example, Beske et al. (2016) were able to establish synaptic activity as a functionally relevant reporter for clostridial neurotoxin (CNT) intoxication through whole-cell patch clamp electrophysiology. The whole-cell patch clamp is achieved by creating a negative pressure that ruptures the cell membrane, allowing for electrical access to the entire cell instead of ion channels exclusively. By measuring the frequency of spontaneous synaptic neurotransmission, Beske’s team effectively reproduced antitoxin protective effects from CNTs botulinum neurotoxin serotypes A and B (BoNT/A and BoNT/B) on mouse embryonic stem cell-derived neurons (ESNs), using the current standard technique mouse lethality assay as a benchmark. With respect to developmental neurotoxicity, this remains a promising and more humane alternative to both mouse and human *in vivo* testing, while being clinically and physiologically relevant.

Another method of tracking neuron functionality can be achieved using calcium indicators. During neurotransmission, an influx of calcium ions into the presynaptic terminal triggers the exocytosis of neurotransmitter-containing vesicles into the synaptic cleft. Tracking the movement of calcium using indicators allows for neuronal signaling to be imaged using high-speed confocal microscopy (Grienberger and Konnerth, 2012). Significant advancements in generating protein-based genetically encoded calcium indicators (GECIs) have led to the development of new methods for calcium imaging *in vivo*, the latest of which have been focused on GCaMPs (Zhang et al., 2023). To study the direct and modulatory synaptic influences on cholinergic myenteric ganglion (MG) neurons, Margiotta et al. (2021) used engineered mice expressing GcaMP6f and biosynthetic choline acetyltransferase (ChAT) selectively in neurons. Among other findings, Margiotta’s team was able to determine that cholinergic MG neurons require sodium channel-dependent impulses in the axonal presynaptic input in order to generate spontaneous action potentials in the postsynaptic soma. This was conducted by comparing the frequency and peak amplitude of Ca^2+^ transients in ChAT^+^/GCaMP6f^+^ MG neurons using tetrodotoxin (TTX), a known neurotoxin that inhibits voltage-gated Na^+^ channel-dependent action potentials (Chen and Chung, 2014). In the realm of DNT testing, various compounds of interest can be introduced and used to determine any changes in Ca^2+^ transients as a result.

Microelectrode arrays (MEAs) are another increasingly popular system for high-throughput functional phenotyping and drug screening applications. Ranging from 12 to 96-well formats, these systems are comprised of tissue culture plates with a grid of small electrodes embedded within the bottom of each well which then allows for the extracellular action potential from cultured neurons to be recorded. There have been various formats used, including for 2D and 3D structures (Shafer et al., 2019; Passaro and Stice, 2021). MEAs can provide insight on network-wide properties, such as connections, and incorporate many benefits of non-invasive patch clamp electrophysiology. Challenges associated with MEAs have revolved around data analysis and interpretation of experimental results, with a team from Finland creating a dataset and analysis tools for others to validate their findings with (Kapucu et al., 2022b; McCready et al., 2022). MEAs have recently been used in testing for DNT hazard by Shafer et al. (2019) utilizing a network formation assay in cortical cells to test 136 unique chemical compounds for developmental neurotoxicity. However, MEAs may not be as sensitive as patch clamp recordings, which could have played a role in Shafer’s team reporting an assay sensitivity (true positives) of 0.78. Regardless, MEAs are a valuable tool, providing high-throughput, robust information that may eventually become the new gold standard of functional assays.

#### 1.3.3 Gene and protein expression

**FIGURE 3:**
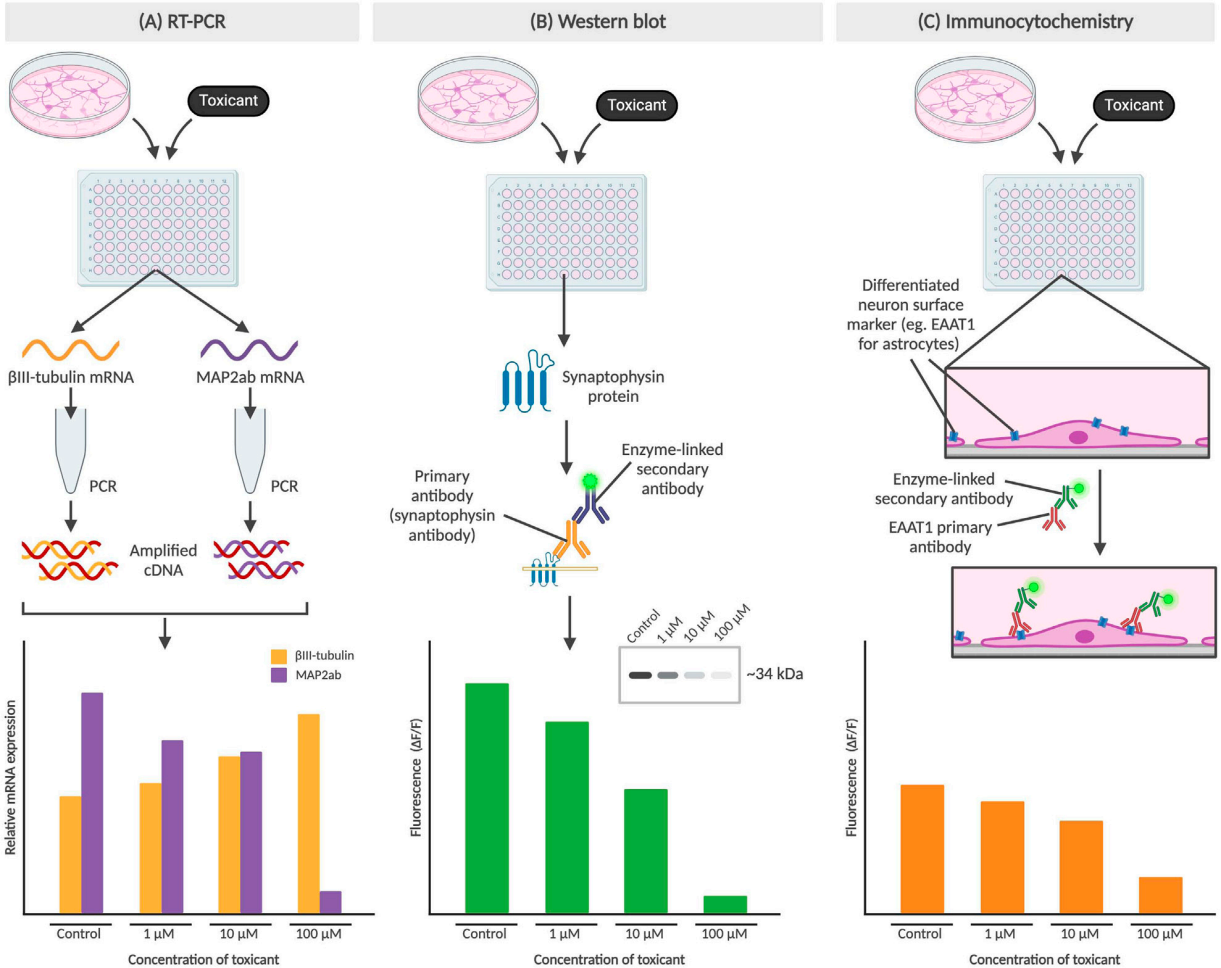
Methods for determining gene or protein expression in neural cells. **(A)** RT-PCR of βIII-tubulin and MAP2ab mRNA from NPCs, with an increase in βIII-tubulin indicating lower concentration of differentiated neurons which may be due to differentiation-inhibiting effect of a toxicant. **(B)** Western blot detects protein levels, such as synaptophysin (presynaptic vesicle membrane protein) and directly correlates to fluorescence of the sample from an enzyme-linked secondary antibody binding to the F_c_ region of the primary antibody. The cytotoxic effect of a toxicant would thus result in a diminished fluorescence level. **(C)** Immunocytochemistry also involves the use of primary and secondary antibodies but preserves cell structure as the primary antibodies bind to surface markers of adhered cells. In the example above, the toxicant results in the prevention of NPC differentiation into astrocytes, which results in a decrease of astrocyte-associated surface marker EAAT1. All graphs above are not based on real data, but represent the conclusions described in published studies.

Gene and protein expression analysis can be carried out to study the impact on NSCs as a result of exposure to drugs or environmental compounds. As shown in Figure 3, researchers can identify cell differentiation, maturity and up/downregulation of the gene or protein of interest using several methods, including reverse transcription polymerase chain reaction (RT-PCR), Western blots and immunocytochemistry.

RT-PCR works by generating cDNA from mRNA, with its high sensitivity allowing for detection of gene expression from a single cell (Bachman, 2013). By comparing gene expression in NSCs pre- and post-exposure to the compound, the effects of a compound of interest can be determined. After exposure to the compound, the mRNA produced is isolated and prepped for RT-PCR. Primers selected to identify key genes for cell fate include βIII-tubulin (Tuj-1) as a marker of immature or early differentiated neurons, and MAP2ab as markers for mature neurons (Soltani et al., 2005; Kermani et al., 2008). These primers will only bind to complementary sequences on the cDNA, amplifying the genes of interest to detectable levels which can then be detected using either gel electrophoresis or primers with fluorescent tags (Kermani et al., 2008).

Western blots and immunocytochemistry are methods used to track changes in protein expression after NSCs are exposed to the compound of interest. Western blots typically utilize a BCA protein assay to first determine overall protein concentration in lysed NSCs, followed by gel electrophoresis to separate proteins and a fluorescent antibody stain to visualize target protein (Engstrom et al., 2015; Hobson et al., 2022). The fluorescence is detected using a confocal microscope and can then be processed to analyze protein expression. For example, synaptophysin, a presynaptic vesicle membrane protein critical for neurotransmission, has been used as a proxy to study the protective effects of physical exercise by a team in Brazil (Leardini-Tristão et al., 2020).

Immunocytochemistry involves fixing post-exposure NSCs typically with a cross-fixing agent such as paraformaldehyde and staining with fluorescent primary and secondary antibodies for specific proteins of interest, before an imaging process similar to that seen in Western blots. While both methods allow for detection of presence of and relative abundance of the protein of interest, immunocytochemistry is performed on intact NSCs which additionally allows for subcellular localization. EAAT1, or excitatory amino acid transporter 1, is a glutamate transporter found on the surface of astrocytes and plays a role in glutamate reuptake from the synaptic cleft (Malik and Willnow, 2019). As the main excitatory system, astrocytic EAAT1 levels gradually become upregulated during CNS development and therefore can provide insight on the inhibitory effects of toxicants, particularly on synaptic transmission (Araque et al., 1998; Banner et al., 2002).

#### 1.3.4 Morphology

**FIGURE 4:**
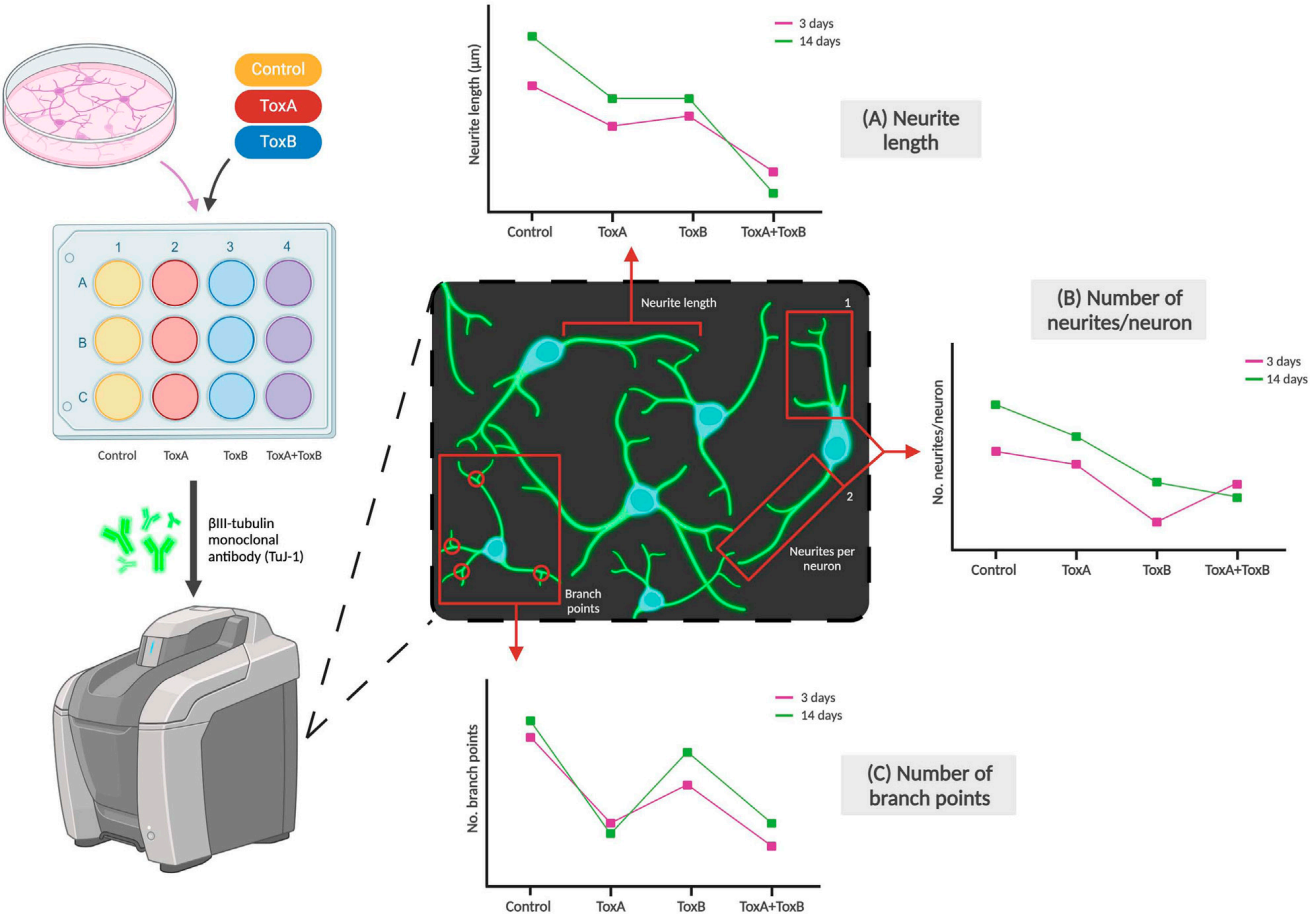
Examples of morphological changes of neurons due to exposure to toxicants. Neuronal markers like βIII-tubulin can be used to stain neurites which can then be imaged using a fluorescent microscope. The effects of toxicant type (examples labeled ToxA and ToxB above) and dose on **(A)** Neurite length, **(B)** Number of neurites per neuron, and **(C)** Number of branch points per neuron can be observed. The graphs are not based on real data but are adapted by Pistollato et al. and represent some of the team’s conclusions (Pistollato et al., 2020).

The effect of neurotoxicants on NSCs or neurons can be determined based on their morphology, such as changes in cell size or fragment length, branches, and total length per cell. These morphological changes can indicate the impact of the toxicant on cellular differentiation and cytotoxicity as shown in Figure 4. Crumpton et al. (2001) used a morphological index to identify the most sensitive period during differentiation for which the toxicant had the greatest effects on the stem cells, concluding that lead had the greatest effect during the early initiation events of differentiation. Investigating morphological changes provides a straightforward and effective method for determining impacts of a compound of interest on the physical characteristics of neural cells. In a study conducted by Pistollato et al. (2020) on developmental neurotoxicity, one of the DNT-specific endpoints assessed was neurite outgrowth which was analyzed using β-III-tubulin staining. The team concluded that a combination of chemicals which they defined to have similar modes of action [i.e., Bisphenol A (BPA), Lead (II) chloride (Lead), and Chlorpyrifos (CPF)] can produce higher levels of toxicity compared to single chemicals, as seen in downregulated neurite length and number of branch points compared to each of the single chemicals (Pistollato et al., 2020).

### 1.4 Challenges with current methods

The assays mentioned above utilize different principles to assess the *in vitro* effects of potential DNTs. However, there are challenges faced by each of the methods as summarized in Table 1 which can affect their ability to accurately determine the full extent of the effects of each compound or toxicant. These challenges can be overcome by employing several techniques over the course of the investigation (Seal et al., 2021; Seal et al., 2022). However, there are certain limitations shared across all of the methods above which involve cell culture type (2D and 3D) and screening efficiency (LTS vs. HTS).

**TABLE 1:** Description and challenges associated with assays used to evaluate potential DNTs.

Method	Description	Challenges
Cytotoxicity and cell viability	Based on cell metabolism or intact cell membrane	Dyes can interact with non-target compounds, leading to false attributions and overestimation of cell viability (Braun et al., 2018)
Functionality	Based on calcium influx or action potential generation	Limited by kinetics and signal-to-noise ratio Functional synapses are preserved in slice preparations, which cannot be used for high-throughput automated patch clamp Current calcium indicators inadequate at studying rapid timescales relevant to neural behavior (Zhang et al., 2023)
Gene and protein expression	Based on changes in cell gene and protein expression	Potential non-specific amplification when low or no gene expression (Mitchell and Iadarola, 2010)
Morphology	Based on changes in cell’s physical features	NSCs dynamic growth and development can cause difficulty in determining significant differences against baseline (Schaudien et al., 2018)

#### 1.4.1 2D and 3D cell cultures

Methods used to evaluate developmental toxicants have conventionally been conducted in 2D cultures. With a simple culture process and low-cost maintenance, 2D cultures provide a reproducible method of conducting functional tests that are relatively high-performing for their cost. However, there are also several drawbacks to adherent cell cultures. Neural cells cultured on a flat surface may be unable to mimic the organizational complexity and structure of brain tissue, and cellular interactions are limited to peripheral contact. This disadvantage impacts certain aspects of *in vivo* modeling, as crucial cell-cell and cell-extracellular matrix interactions that are responsible for cell differentiation and proliferation, survival, and morphology may not be as easily replicated (Brännvall et al., 2007a; Xu et al., 2009; Pontes Soares et al., 2012; Centeno et al., 2018). Monolayer cultures also have access to unlimited nutrients, oxygen, and other essential metabolites as all cells are in direct contact with the medium. This can be less representative of *in vivo* conditions, leading to another issue with respect to the evaluation of neurotoxicants. Some studies have demonstrated that 2D cell cultures are more susceptible to the effects of drugs, due to reasons such as exposed receptors enhancing the binding efficacy of drugs (Cushing and Anseth, 2007; Bonnans et al., 2014; Lv et al., 2017; Langhans, 2018). As such, results of neurotoxicant testing in adherent cell cultures may not always be fully representative of the actual effects *in vivo*.

For mechanisms that cannot be as effectively modeled using 2D cultures, researchers have opted to investigate these mechanisms using 3D cultures. There are several approaches used to create 3D cultures, including self-assembling cell aggregation in spheroids/organoids, cell encapsulation, or direct cell plating in either natural or synthetic hydrogels (Huang et al., 2012; Białkowska et al., 2020; Lam et al., 2020; Vallejo-Giraldo et al., 2020). Hydrogels confer a significant advantage to 3D cultures as hydrogels provide a complex network of proteins and cross-linking polymers which mimics that of the extracellular matrix (ECM), facilitating cell-ECM interactions for improving cellular function and morphology. This is especially important for functional assays, which evaluate neural activity, as this is highly dependent on effective cell-cell communication (Lam et al., 2020).

However, there are several disadvantages that 3D cultures face which are actively being addressed as they become more widely used for toxicology studies. Low optical transparency for imaging due to culture density or scaffold limitations can lead to challenges within observing changes in cells exposed to toxicants (Hopkins et al., 2015; D’Avanzo et al., 2015). This is especially important for calcium imaging and characterization of neuronal activity. There is also potentially decreased reproducibility as a result of variability across batches of scaffold material, such as hydrogel (Tibbitt and Anseth, 2009; Caliari and Burdick, 2016; Baruffaldi et al., 2021). The need for expensive and highly specialized equipment, such as bioreactors, to grow and maintain cell cultures also remains a significant barrier to the mainstream use of 3D cultures for DNT testing (Centeno et al., 2018). While there are still challenges towards the widespread implementation of 3D cultures in research, it is becoming an increasingly popular choice. 3D culture platforms offer a robust way to culture cells and study their cellular functions, and thus represent a new modality to complement 2D cultures in understanding the effect of compounds on the differentiation of stem cells to early neurons. The use of 2D or 3D cultures within neurotoxicant testing ultimately remains up to the researcher, with both providing different information relevant to *in vivo* behavior and each type of culture having their own advantages and disadvantages.

#### 1.4.2 LTS vs. HTS

One strategy of optimizing and improving the cost effectiveness of manufacturing cell cultures has been through high-throughput screening (HTS). HTS platforms commonly use hydrogel microarrays to study the effects of numerous physical and biochemical properties on cell behavior, allowing for the production of large-scale reproducible results while minimizing use of reagents and samples (Seo et al., 2018). This can be implemented in both 2D and 3D cultures, with a recent protocol for the generation of 3D spheroids from NSCs meant for the use in HTS published in 2022 (Kang et al., 2021). However, there has been some variability in effectiveness of both types of cultures in HTS. Lu et al. (2023) developed both 2D and 3D microarray platforms in 384-well plates to detect neurotoxicants through studying their effects on human induced pluripotent stem cell (hiPSC)-derived cultures. The team introduced 25 reference drugs with known effects on human seizure risks, and through blind testing were able to determine a 91% predictive accuracy in determining seizure-inducing neurotoxicants within 2D cultures, and a 45% accuracy in 3D cultures. Their data suggests that while there seems to be reliable prediction of DNTs in 2D cultures, there needs to be further investigation into optimizing HTS methods in 3D cultures.

While the differences may have been a result of limitations mentioned in the previous section, another explanation could be the type of 3D culture used. Spheroids, which were used by Lu et al. (2023), are considered to be lower complexity with less physiological relevance. On the other hand, organoids are heterogeneous aggregates that partially resemble the organ in both structure and function (Renner et al., 2020). HTS using brain organoids can potentially alleviate this issue, however generating fully matured organoids can take a significant amount of time – anywhere from 20 days to 6 months depending on the type of neuron (Renner et al., 2020; Woodruff et al., 2020; Porciúncula et al., 2021). Renner et al. (2020) addressed this long pre-processing time by developing a protocol using an automated liquid handling system to culture and phenotype midbrain organoids, highlighting the potential for improvements in this field through automation.

Nonetheless, even with improvements to 3D culture generation and scale-up, there are still issues that remain within the acquisition, extraction, and processing of large quantities of data from HTS methods. Light scattering which limits the penetration depth of light (roughly 50–70 μm) into 3D cultures for conventional imaging leads to noisy datasets that require careful clean-up and analysis to provide any meaningful interpretations (Wang and Jeon, 2022). To address this, machine learning algorithms (MLAs) are becoming an increasingly popular choice to handle large and complex datasets. MLAs can also utilize multidimensional analysis of various parameters which would allow for a more holistic interpretation of several factors to the target cell culture (Lin et al., 2020). A shift towards integrating machine learning, such as artificial intelligence, are becoming increasingly common within drug screening. As such, there is a call for the integration of predictive models that could better inform testing batteries for DNTs. Currently, predictive models are employed for several types of studies, including determining cell fate of NSCs. Understanding these novel models can allow researchers to gain more insight into how these models can then be applied for use in screening potential DNTs.

## 2 Predictive models for neuronal cell fate

### 2.1 What are they and how are they used?

Predictive models use existing data to make predictions on the outcome of a new unknown input given defined model parameters. In neural stem cell (NSC) systems, data on NSC response to exogenous factors (which includes both test system environment or substances) may be quantitatively analyzed, developing computational models that can then be used to predict or even control stem cell fate (Viswanathan and Zandstra, 2003). These algorithms obtain parameters by fitting their models to experimental data studying net changes in NSC populations using different variables.

There are two main types of predictive models: Data-driven and mechanistic models. Data-driven, or empirical, approaches are typically employed for pattern recognition, classification, and prediction, establishing relationships between extensive amounts of input data with the predicted output (del Sol and Jung, 2021). On the other hand, mechanistic models use mathematical formulations that describe the biological or physical mechanisms that occur in a system. To create a mechanistic model, the cellular mechanisms (such as signal transduction pathways, receptor binding affinity, etc.) must be defined and understood so as to model and predict a compound’s effect (van der Graaf, 2018; Collin et al., 2022). It should also be mentioned that while data-driven and mechanistic models operate using two different methodologies, they can be complementary approaches to improve efficiency and optimize predictive power. As an example, NSCs undergo differentiation and self-renewal in culture and as such both differentiation and proliferation of individual NSC populations to be examined simultaneously while altering environmental conditions (Morrison, 2001). In this case, mechanistic models could serve as a straightforward and effective tool to discern the effects of exogenous factors, ranging from toxicants to substrate surface, on NSC fate decisions, with data-driven machine learning used to filter and sort the input going into the mechanistic model.

### 2.2 Cost and efficiency

Even with the advancements made in the field, there remains a significant barrier to implementing robust screening batteries for DNTs. For the approaches described above, the cost and efficiency of such methods are still an issue, especially considering the highly involved nature of developing such strategies. This issue is not unique to toxicology screenings. Within the context of drug discovery, conventional methods include HTS using large libraries to identify potential “hit compounds,” which are then experimentally tested for biological effectiveness on a target ligand. With the cost of screening one potential compound sometimes upwards of $1.50 per well, Dreiman et al. (2021) found that employing an iterative screening model using machine learning could improve rates of finding hit compounds which would then optimize their experimental testing. Another team used virtual libraries to eliminate compounds with known toxic or reactive functional groups to narrow the experimental space (Zhu et al., 2013). Similarly, utilizing an *in silico* approach coupled with downstream experimental testing may open up complementary strategies for informing future DNT experimental testing – using frameworks such as the integrated approaches to testing and assessment (IATA) (Bal-Price et al., 2018; Hernández-Jerez et al., 2021; OECD, 2023). These can also significantly reduce the cost and amount of resources expended during testing.

### 2.3 Advantages

Predictive modeling serves as an alternative to experimental tests difficult to perform, due to reasons such as the high cost and time constraint for a large number of trials. Since predictive models use existing data to determine causations or correlations, they can reliably inform of outcomes given inputs in several different parameters, allowing for simultaneous analysis of multiple factors to influence cell fate. Generating any accurate model requires large quantities of experimental data, standardized and normalized to account for any variations across samples. Significant advancements in single cell transcriptomics, or scRNA-seq, have led to higher accuracy in detailing the processes which lead to stem cell differentiation. Along with other methods to classify single cells and their eventual cell fate, the growing amount of reliable existing data can make predictive models even more robust in their predictions (Alquicira-Hernandez et al., 2019; Sagar and Grün, 2020).

### 2.4 Predictive modeling for neural progenitor cell fate

There are numerous factors which affect the behavior of NSCs, especially concerning their cell fate. Some of these include material morphology and chemical molecules, which can be studied in more detail to predict outcomes beyond what is tested in the lab (Huang and Wang, 2017). This can provide more robust and efficient predictions that allow for the identification of new protocols to induce proliferation and differentiation towards a target cell type. To develop predictive models for neural progenitor cell fate, research teams have explored various cell conditions such as culture type (2D or 3D), presence of chemical toxicants, and substrate stiffness, among others. Being a relatively established field of study, these predictive models have been validated against existing data which can ultimately serve as a frame of reference for the development of novel predictive models for DNT testing.

#### 2.4.1 2D or 3D

It has been established that cell cultured on 3D cell matrices have a higher rate of neural stem cell differentiation, along with increased cell survival and proliferation (Brännvall et al., 2007b; Ortinau et al., 2010; Zare-Mehrjardi et al., 2011; Huang et al., 2013; Bozza et al., 2014). With respect to neural cell differentiation, these studies looked at gene expression profiles for common neuronal markers that indicate differentiation and maturation, such as TUBB3 (for βIII-tubulin) or MAP2 (for MAP2ab). In another application, Zhu et al. developed a deep learning platform that could predict an NSC’s eventual cell differentiation into neurons, astrocytes, or oligodendrocytes through monitoring the gene expression levels of NeuN, GFAP, and Olig2 respectively (Zhu et al., 2021). With a spectrum for the functionality and formation of a 3D microenvironment using hydrogel scaffolds, a predictive model can be designed to determine how successful different types of hydrogel-based scaffolds can influence cell differentiation of NSCs by tracking the expression of various markers such as those mentioned above.

#### 2.4.2 Chemical toxicants

The presence of certain chemical compounds can influence cell differentiation, proliferation, and cell apoptosis of NSCs (Huang et al., 2019). While investigating the effects of neurotoxicants in particular, understanding the dose-response relationship along with duration of exposure are important factors to consider. In one instance, Lee et al. (2018) identified a toxicity response gene, SERPINB2, which is overexpressed in stem cells both *in vitro* and *in vivo* upon exposure to dioxin. Lee’s team noted decreased levels of self-renewal and differentiation potential of stem cells during the exposure, suggesting that SERPINB2 would be a good candidate as a stem cell toxicity marker. In this case, evaluating potential drug candidates for toxicity could involve quantifying levels of SERPINB2 expression pre- and post-exposure. Similarly, Mao et al. (2022) incorporated *in vitro* toxicity profiles to develop an *in silico* platform to understand and predict toxicity patterns of silver nanoparticles (AgNPs). They began by introducing several mammalian cell lines to four types of fabricated AgNPs: citrate-coated (SCS), cysteamine-coated (SAS), citrate-coated (LCS), and cysteamine-coated (LAS). Cell viability assays were used to generate data that was then consolidated into a database with several cytotoxicity-relevant parameters such as cell type and exposure dose. The model utilizes the assay data to then identify which parameters are most important in predicting AgNPs induced toxicity. One prediction, determining that exposure time was the least influential factor on the cytotoxicity profile of AgNPs, directed the authors to then investigate and ultimately conclude that non-cytotoxic doses of AgNPs potentially resulted in autophagy and senescence in cells.

#### 2.4.3 Stiffness

Stem cells are highly sensitive to mechanical inputs – such as stiffness – from the ECM and this is known to influence subsequent differentiation and proliferation (Engler et al., 2006; Pek et al., 2010; Her et al., 2013). Based on a paper by Engler et al. (2006) it was identified that in general naive mesenchymal stem cells (MSCs) differentiated on a substrate with matrix stiffness between 0.1–1 kPa expressed neurogenic markers, 8–17 kPa expressed myogenic markers, and 25–40 kPa expressed osteogenic markers. Substrate stiffness and topography are also known to impact both proliferation and differentiation of NSCs towards several cell types such as oligodendrocytes, astrocytes, and neurons (Banerjee et al., 2009; Leipzig and Shoichet, 2009; Ali et al., 2015; Jiang et al., 2015; Rammensee et al., 2017; Stukel and Willits, 2018; Liang et al., 2021; Mattiassi et al., 2023). In order to reliably predict cell fate based on surface topography, Heydari et al. (2017) designed a virtual cell model which included substrate characteristics such as stiffness. The team was able to obtain qualitative data surrounding the behavior of MSCs and their interactions with their ECM, with one example being the mimicking of substrate shape. Their work also demonstrated the potential of predictive models like the virtual cell to streamline the identification of optimal substrate compositions for a specific cell fate (Heydari et al., 2017).

### 2.5 How may they be used for predictive neurodevelopmental toxicity?

**FIGURE 5:**
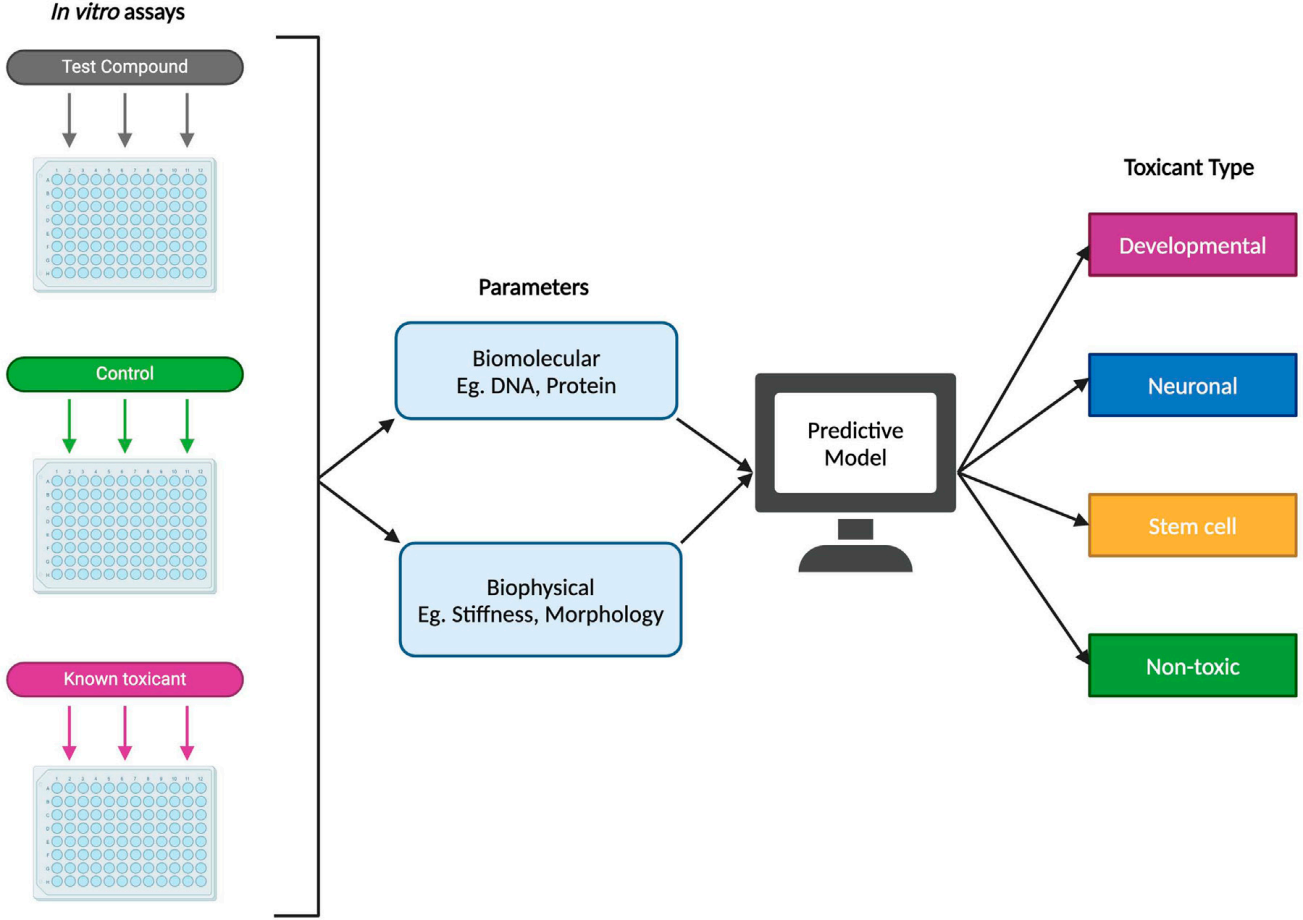
Example of a proposed predictive model for developmental neurotoxicity using *in vitro* experimental data. Using both biomolecular and biophysical parameters, a proposed predictive model could potentially classify a novel test compound into one of several types: developmental (specific detrimental effects during fetal development), neuronal (affecting differentiated neural cells), stem cell (targeting stem cells or progenitor cells), or non-toxic.

Predictive models and machine learning have the potential to serve as reliable, efficient, and high-throughput approaches to identifying possible DNTs. That is not to say that these methods should act as a replacement of *in vitro* and *in vivo* studies, but as a complementary tool that can reduce the experimental space for such studies. When it comes to predictive toxicology, *in silico* modeling has already shown great promise in identifying toxicants already confirmed through *in vitro* and *in vivo* methods (Knudsen et al., 2020; Zurlinden et al., 2020). This may be combined with computational embryology, which can help to make inferences about a potential DNT’s effect during prenatal development as such information may not be possible to derive experimentally (Knudsen et al., 2020). One proposed method of designing a model for predictive developmental neurotoxicity is illustrated in Figure 5 and includes using biomolecular and biophysical endpoints and data from *in vitro* studies, such as cell proteomics and stiffness respectively, to determine the type of toxicant being introduced into the system (developmental, neuronal, or stem cell toxicant, etc.). In this proposed method, toxicant type could potentially refer to which stage of differentiation the toxicant produces a detrimental effect, as it has been estimated in one study that certain compounds may lead to cytotoxic or other negative impacts if introduced at specific time points during the neurodevelopmental process (Heyer and Meredith, 2017). In another study, Pei et al. (2016) reported that 40%, 47.5%, 57.5%, and 51.3% from a set of 80 tested compounds produced significant cytotoxicity in iPSCs, NSCs, neurons, and astrocytes respectively – with the fewest number of compounds being significantly cytotoxic in iPSCs. The results from this study suggest that there may be merit in differentiating toxicant types based on the type of cells it affects, as some compounds impacting fully differentiated cells like astrocytes or neurons may not have a significant inhibitory effect on their parent cells such as iPSCs or NPCs.

Strategies employed to inform drug development, like physiologically based pharmacokinetic models (PBPK) can also provide promising applications within toxicity studies through improving *in vitro* to *in vivo* extrapolation (IVIVE) (Zhou et al., 2021). For example, Singh et al. (2020) developed a PBPK model integrated with pharmacodynamics (PD) which allowed for the characterization of their CAR-T cells to better understand their clinical behaviors like efficacy and toxicity. Another concept important to highlight is the adverse outcome pathway (AOP) model. As defined by the National Toxicology Program, AOPs “[identify] the sequence of molecular and cellular events required to produce a toxic effect when an organism is exposed to a substance.” (U.S. Department of Health and Human Services, 2023) These can provide a conceptual framework to standardized toxicity testing methods, combining data generated from relevant *in vitro* methods to map them towards endpoints like developmental toxicity. In an effort to methodically develop predictive models for DNT testing, AOPs are a powerful tool that can allow for accurate prediction of downstream response and effects of toxins and toxicants on both the molecular and cellular level (Pistollato et al., 2020). An example of an AOP can be seen in Table 2, where inhibition of the vascular endothelial growth factor receptor (VEGFR) results in a sequence of effects that ultimately lead to developmental defects in an individual (Knudsen et al., 2023).

**TABLE 2:** Example of an AOP: Disruption of VEGFR signaling leading to developmental defects (AOP 43) available from https://aopwiki.org/aops/43 (Knudsen et al., 2023).

Level of Organization	AOP diagram (Knudsen et al., 2023)	Event
Macromolecular	Inhibition of VegfR2	Molecular initiating event (MIE): specialized key event that triggers AOP
Cell/Tissue	Reduction of angiogenesis	Key events (KEs): measurable event within specific level of biological organization
Organ/Organ system	Impairment of endothelial network	
	Vascular insufficiency	
Individual	Increased developmental defects	Adverse Outcome (AO): key event of regulatory significance that concludes an AOP

## 3 3D developmental neurotoxicity predictive models

### 3.1 Morphology

Morphology-based predictive models have been proposed for various applications such as classifying and predicting cell health phenotypes, along with determining cancer cell migration patterns (Zhang et al., 2018; Li et al., 2021; Way et al., 2021). These models typically use single-cell image feature extraction of *in vitro* data to categorize and validate which morphological properties are important for their target prediction output. Predictive models are especially useful for assessing stem cell differentiation as traditional methods (RT-PCR, immunostaining, etc.) are time-consuming, expensive, and may face challenges due to the dynamic nature of stem cell growth. Several teams have been able to successfully develop morphology-based models to predict both adipogenic and osteogenic differentiation from MSCs (Lan et al., 2022; Mai et al., 2023).

In 2022, the USEPA’s Center for Computational Toxicology and Exposure (CCTE) published a presentation by Megan Culbreth, PhD, detailing a HT phenotypic profiling strategy that looks at chemically-induced changes in human neural progenitor cell morphology specifically for DNT hazard assessment. Based on a paper published by Culbreth et al. (2022) in *Frontiers*, the model described was able to identify cell-level features like nuclei, nucleoli and ER, golgi and plasma membrane, and mitochondria separately. Using these parameters, a phenotypic profile can then be generated and applied to compare the effects of the target compound against profiles generated by reference chemicals.

However, one drawback of such assays is that DNTs may affect critical processes of nervous system development that cannot be reproduced by neural progenitor cells or other stem cell models. For example, neuronal morphology plays a large role in dictating the architecture of the cortical network. While axo-dendritic overlaps are necessary to the formation of synapses, several other properties can more reliably predict the nonrandom occurrence of network motifs. This includes cell diversity, dendrite polarity, or geometric features like packing density (Gal et al., 2020; Udvary et al., 2022). Interfering with such delicate mechanisms (like synapse formation) is enough to cause severe and irreversible effects on the developing brain, and single cell morphological changes identified to indicate cytotoxic activity may be unable to establish the detrimental effects of a DNT in this context. As such, moving towards predictive models that include impacts of morphology on the structure and function of features like neocortical synapses may thus provide holistic insights on the true effects of a potential DNT during hazard assessments.

### 3.2 Cell viability and proliferation

Cell viability, in both drug discovery and toxicity studies, is typically assessed through *in vitro* tests like the WST, MTT, or L-Lactate dehydrogenase (LDH) assays. LDH is a cytoplasmic enzyme involved in glycolysis and its quantification in serum has been used clinically to determine the extent of tissue damage, both acute and severe (Kaja et al., 2017). Monitoring proliferation is also important as there may be disruptions that do not directly or immediately lead to cell death but instead impedes growth rate. An example of cell viability and proliferation being a key assessment endpoint is highlighted by Coccini et al. (2020) using magnetite nanoparticles (Fe_3_O_4_NPs). The team first induced transdifferentiation of human umbilical cord MSCs into neurons before tracking changes in cytoplasm retraction and long bipolar cellular processes, typical indicators of the neuronal phenotype, upon the introduction of Fe_3_O_4_NPs. While there were no obvious morphological changes, there was a significant decrease in cell density while Fe_3_O_4_NPs accumulated within the cytoplasm of neuronal-like cells (hNLCs). The team also looked at changes in membrane integrity, adenosine triphosphate and caspase 3/7 activity as proxies for cell death. Ultimately, the team’s findings were consistent with previous studies establishing critical concentrations of Fe_3_O_4_NPs in animal models (Wang et al., 2010; Wu et al., 2013).

Advancements within this space have allowed researchers from the Eunice Kennedy Shriver National Institute of Child Health and Human Development (NICHD) to design a label-free, non-invasive, and repeatable assay using supervised machine learning techniques to assess cell viability (Park et al., 2022). Using dynamic full-field optical coherence microscopy (DFFOCM), the team obtained raw images of cell samples with a frame rate of 300 Hz which were used to calculate two indicators of intracellular movement identified as f_mean_ and magnitude in a prior study done by the team (Park et al., 2021). This data was then fed into four trained algorithm models to indicate live and dead cells at various time points, with a final balanced accuracy of 93.92% ± 0.86%. Such a system is promising in moving the field towards non-invasive methods for determining cell viability and proliferation, which almost certainly will serve to improve the efficiency of existing HT screening methods.

In their initial recommendations for evaluating data from DNT *in vitro* testing batteries (IVBs), the OECD has also released guidelines on how to apply prediction models when analyzing cell viability and proliferation data (OECD, 2023). Some examples of this include assessment of cell proliferation in human neural progenitor cells (NPC1) in Appendix B.1 and the high-content imaging assay screening changes in human neuroprogenitor cell (hNP1) upon chemical exposure in Appendix B.10 of the OECD DNT-IVB document. Like all types of *in vitro* data, data collected from these hazard assessment assays require extrapolation towards *in vivo* exposure levels to become clinically relevant. However, the assays have included varying amounts of IVIVE modeling and data – with some stating it has not yet been determined nor well established – demonstrating the experimental gaps that need to be addressed as the OECD continues to improve their guidelines for DNT-IVBs. With specific biomarkers indicating cell proliferation or apoptosis, such as GFAP, MCM2, or p53, cell viability and proliferation can also be output parameters of prediction models (Zhang and Jiao, 2015; Aubrey et al., 2018). Using gene and protein expression profiles, prediction tools which efficiently generate hypotheses to characterize potential chemical toxicants for exposure-based assessments have been designed (Zurlinden et al., 2020).

### 3.3 Gene or protein expression

Gene and protein expression profiles can be used as proxies for tracking changes in various cellular characteristics and physiological processes – such as proliferation, differentiation, and apoptosis. These can be seen to form a unique “fingerprint” indicating the cell’s current physiological state. For example, neural markers such as β-Tub III, MAP-2, NSE, and nestin have varying expression levels during the differentiation process of hNLCs allowing researchers to approximate which stage the cells are in (Coccini et al., 2020). In another paper, Lauvås et al. (2022) determined the impact of acrylamide – and its metabolite glycidamide – on fundamental neurodevelopmental processes by studying both gene and protein expression in a 3D mixed culture of neurons and astrocytes. With the dynamic nature of the differentiation and maturation processes, time course experiments can be used to generate baseline gene expression profiles used for predictive modeling – with any deviation from these profiles potentially indicating an inhibition of cell differentiation or growth.

Some teams have also been able to use existing gene expression databases to develop machine learning models that can predict drug toxicity. One team from Shanghai Ocean University developed a cell viability prediction algorithm that can predict cell viability upon administration of various drugs or shRNA using different high-coverage molecular data recovered from six different databases, including the perturbation transcriptomics signatures (LINCS-L1000) and Cancer Therapeutics Response Portal (CTRP) (Lu et al., 2021). Similarly, Zurlinden et al. (2020) designed a predictive model that utilized relative changes in the ratio of two metabolites of cellular respiration, ornithine and cystine (ORN/CYSS), to predict a substance’s potential developmental toxicity. Using the Human-Mouse Disease Connection (HMDC) database with established gene-to-disease associations, Zurlinden’s team isolated genes with a biological relevance before correlating the genotypes to specific biochemical features in the ToxCast NovaScreen assay.

## 4 Conclusion

No individual NAM or AOP covers all key neurodevelopmental biology or processes, meaning that a battery of tests are required to ensure rigorous DNT testing. With large amounts of data, predictive models can serve as an efficient tool to quickly discover correlations that allow for the identification of potential DNTs. In a paper detailing a proposed cell-based IVB for DNT testing, the use of predictive models in this space is still considered an open-ended question for many researchers (Blum et al., 2023). It is important to note that while DNT predictive models have certain limitations in their specificity and accuracy, they still provide value by flagging compounds and prioritizing chemicals with potential human toxicity for further evaluation. This can narrow down the scope of experimentation to optimize investigations towards other aspects, such as estimated adverse doses (AEDs). Instead of aiming to fully replace animal and *in vitro* testing, the shift towards *in silico* methods serves to both complement and reduce reliance on such methods – especially considering the assay gaps for numerous key neurodevelopmental processes in the current DNT-IVB as seen in Table 3 (Masjosthusmann et al., 2020; OECD, 2023).

**TABLE 3:** Summary of key neurodevelopmental processes and DNT-IVB assay gaps (due to lack of complementary assays) (Masjosthusmann et al., 2020; OECD, 2023).

Key Neurodevelopmental Process	Gaps in Current DNT-IVB
Neural Progenitor Cell (NPC) Proliferation	Human-induced pluripotent stem cells (hiPSC)-derived NPCs Radial glia NPCs
NPC Apoptosis	Complementary assays in place
Cell Migration	Complementary assays in place
NPC Neuronal differentiation	Neuronal subtype Human-induced pluripotent stem cells (hiPSC)-derived NPCs
Neurite outgrowth	Complementary assays in place
Neurite maturation	Human neuron
Synaptogenesis	Human neuron
NPC Glial differentiation	Astrocytes Radial glia
Myelination	Oligodendrocytes
Neural network formation	Human neuron-based

In their latest recommendations on the evaluation of DNT-IVB data, the OECD (2023) notes that there has been a paradigm shift away from *in vivo* animal tests towards *in vitro* models and higher throughput technologies within DNT testing. The OECD also states that DNT-IVB testing should be guided by a problem formulation approach driven by regulatory needs and acceptability using the IATA framework, a recommendation supported by the European Food Safety Authority (EFSA) back in 2021 (Hernández-Jerez et al., 2021; OECD, 2023). Currently, the OECD is focused on developing a tiered testing strategy system for DNT that can allow for a broad range of guidelines in the context of *in silico*, *in vivo*, as well as IVIVE applications (OECD work on *in vitro*). Such an approach would include Tier one preliminary screenings for potential developmental neurotoxicity, following up any “hits” with additional, more intensive Tier two testing (Becker et al., 2007; Giordano and Costa, 2012). Recognition by regulatory bodies like the US EPA, EFSA, and OECD through their guidelines play an important role in establishing standards for new developments within areas like developmental neurotoxicity. While these guidelines can only serve as a form of guidance rather than regulation, they allow researchers to more comprehensively understand the capabilities of predictive modeling for developmental neurotoxicity testing, leading to advancements throughout the field.

## References

[B1] Abbott L. C. Nigussie F. (2021). Mercury toxicity and neurogenesis in the mammalian brain. Int. J. Mol. Sci. 22 (14), 7520. 10.3390/ijms22147520 34299140 PMC8305137

[B2] Ali S. Wall I. B. Mason C. Pelling A. E. Veraitch F. S. (2015). The effect of Young's modulus on the neuronal differentiation of mouse embryonic stem cells. Acta biomater. 25, 253–267. 10.1016/j.actbio.2015.07.008 26159105

[B3] Alquicira-Hernandez J. Sathe A. Ji H. P. Nguyen Q. Powell J. E. (2019). scPred: accurate supervised method for cell-type classification from single-cell RNA-seq data. Genome Biol. 20 (1), 264. 10.1186/s13059-019-1862-5 31829268 PMC6907144

[B4] Araque A. Parpura V. Sanzgiri R. P. Haydon P. G. (1998). Glutamate-dependent astrocyte modulation of synaptic transmission between cultured hippocampal neurons. Eur. J. Neurosci. 10 (6), 2129–2142. 10.1046/j.1460-9568.1998.00221.x 9753099

[B5] Aschner M. Allen J. W. Kimelberg H. K. LoPachin R. M. Streit W. J. (1999). Glial cells in neurotoxicity development. Annu. Rev. Pharmacol. Toxicol. 39, 151–173. 10.1146/annurev.pharmtox.39.1.151 10331080

[B6] Aubrey B. J. Kelly G. L. Janic A. Herold M. J. Strasser A. (2018). How does p53 induce apoptosis and how does this relate to p53-mediated tumour suppression? Cell death Differ. 25 (1), 104–113. 10.1038/cdd.2017.169 29149101 PMC5729529

[B7] Bachman J. (2013). Reverse-transcription PCR (RT-PCR). Methods Enzym. 530, 67–74. 10.1016/B978-0-12-420037-1.00002-6 24034314

[B8] Bal-Price A. Pistollato F. Sachana M. Bopp S. K. Munn S. Worth A. (2018). Strategies to improve the regulatory assessment of developmental neurotoxicity (DNT) using *in vitro* methods. Toxicol. Appl. Pharmacol. 354, 7–18. 10.1016/j.taap.2018.02.008 29476865 PMC6095942

[B9] Banerjee A. Arha M. Choudhary S. Ashton R. S. Bhatia S. R. Schaffer D. V. (2009). The influence of hydrogel modulus on the proliferation and differentiation of encapsulated neural stem cells. Biomaterials 30 (27), 4695–4699. 10.1016/j.biomaterials.2009.05.050 19539367 PMC2743317

[B10] Banner S. J. Fray A. E. Ince P. G. Steward M. Cookson M. R. Shaw P. J. (2002). The expression of the glutamate re-uptake transporter excitatory amino acid transporter 1 (EAAT1) in the normal human CNS and in motor neurone disease: an immunohistochemical study. Neuroscience 109 (1), 27–44. 10.1016/s0306-4522(01)00437-7 11784698

[B11] Baruffaldi D. Palmara G. Pirri C. Frascella F. (2021). 3D cell culture: recent development in materials with tunable stiffness. ACS Appl. bio Mater. 4 (3), 2233–2250. 10.1021/acsabm.0c01472 35014348

[B12] Becker R. A. Plunkett L. M. Borzelleca J. F. Kaplan A. M. (2007). Tiered toxicity testing: evaluation of toxicity-based decision triggers for human health hazard characterization. Food Chem. Toxicol. Int. J. Publ. Br. Industrial Biol. Res. Assoc. 45 (12), 2454–2469. 10.1016/j.fct.2007.05.030 17689851

[B13] Bell D. C. Dallas M. L. (2018). Using automated patch clamp electrophysiology platforms in pain-related ion channel research: insights from industry and academia. Br. J. Pharmacol. 175 (12), 2312–2321. 10.1111/bph.13916 28622411 PMC5980593

[B14] Beske P. H. Bradford A. B. Grynovicki J. O. Glotfelty E. J. Hoffman K. M. Hubbard K. S. (2016). Botulinum and tetanus neurotoxin-induced blockade of synaptic transmission in networked cultures of human and rodent neurons. Toxicol. Sci. official J. Soc. Toxicol. 149 (2), 503–515. 10.1093/toxsci/kfv254 PMC4751230 26615023

[B15] Białkowska K. Komorowski P. Bryszewska M. Miłowska K. (2020). Spheroids as a type of three-dimensional cell cultures-examples of methods of preparation and the most important application. Int. J. Mol. Sci. 21 (17), 6225. 10.3390/ijms21176225 32872135 PMC7503223

[B16] Blum J. Masjosthusmann S. Bartmann K. Bendt F. Dolde X. Dönmez A. (2023). Establishment of a human cell-based *in vitro* battery to assess developmental neurotoxicity hazard of chemicals. Chemosphere 311 (Pt 2), 137035. 10.1016/j.chemosphere.2022.137035 36328314

[B17] Bonnans C. Chou J. Werb Z. (2014). Remodelling the extracellular matrix in development and disease. Nat. Rev. Mol. cell Biol. 15 (12), 786–801. 10.1038/nrm3904 25415508 PMC4316204

[B18] Boyle C. A. Boulet S. Schieve L. A. Cohen R. A. Blumberg S. J. Yeargin-Allsopp M. (2011). Trends in the prevalence of developmental disabilities in US children, 1997-2008. Pediatrics 127 (6), 1034–1042. 10.1542/peds.2010-2989 21606152

[B19] Bozza A. Coates E. E. Incitti T. Ferlin K. M. Messina A. Menna E. (2014). Neural differentiation of pluripotent cells in 3D alginate-based cultures. Biomaterials 35 (16), 4636–4645. 10.1016/j.biomaterials.2014.02.039 24631250

[B20] Brännvall K. Bergman K. Wallenquist U. Svahn S. Bowden T. Hilborn J. (2007a). Enhanced neuronal differentiation in a three-dimensional collagen-hyaluronan matrix. J. Neurosci. Res. 85 (10), 2138–2146. 10.1002/jnr.21358 17520747

[B21] Brännvall K. Bergman K. Wallenquist U. Svahn S. Bowden T. Hilborn J. (2007b). Enhanced neuronal differentiation in a three-dimensional collagen-hyaluronan matrix. J. Neurosci. Res. 85 (10), 2138–2146. 10.1002/jnr.21358 17520747

[B22] Braun K. Stürzel C. M. Biskupek J. Kaiser U. Kirchhoff F. Lindén M. (2018). Comparison of different cytotoxicity assays for *in vitro* evaluation of mesoporous silica nanoparticles. Toxicol. vitro Int. J. Publ. Assoc. BIBRA 52, 214–221. 10.1016/j.tiv.2018.06.019 29940343

[B23] Bu Q. Huang Y. Li M. Dai Y. Fang X. Chen K. (2020). Acrylamide exposure represses neuronal differentiation, induces cell apoptosis and promotes tau hyperphosphorylation in hesc-derived 3D cerebral organoids. Food Chem. Toxicol. 144, 111643. 10.1016/j.fct.2020.111643 32763439

[B24] Caliari S. R. Burdick J. A. (2016). A practical guide to hydrogels for cell culture. Nat. methods 13 (5), 405–414. 10.1038/nmeth.3839 27123816 PMC5800304

[B25] Centeno E. G. Z. Cimarosti H. Bithell A. (2018). 2D versus 3D human induced pluripotent stem cell-derived cultures for neurodegenerative disease modelling. Mol. Neurodegener. 13 (1), 27. 10.1186/s13024-018-0258-4 29788997 PMC5964712

[B26] Chen R. Chung S. H. (2014). Mechanism of tetrodotoxin block and resistance in sodium channels. Biochem. biophysical Res. Commun. 446 (1), 370–374. 10.1016/j.bbrc.2014.02.115 24607901

[B27] Coccini T. Pignatti P. Spinillo A. De Simone U. (2020). Developmental neurotoxicity screening for nanoparticles using neuron-like cells of human umbilical cord mesenchymal stem cells: example with magnetite nanoparticles. Nanomater. Basel, Switz. 10 (8), 1607. 10.3390/nano10081607 PMC7466682 32824247

[B28] Collin C. B. Gebhardt T. Golebiewski M. Karaderi T. Hillemanns M. Khan F. M. (2022). Computational models for clinical applications in personalized medicine-guidelines and recommendations for data integration and model validation. J. personalized Med. 12 (2), 166. 10.3390/jpm12020166 PMC8879572 35207655

[B29] E., Covey, M. Carter, (2015). Basic electrophysiological methods (New York: Oxford Academic), (Accessed April 26, 2023).

[B30] Crofton K. M. Mundy W. R. (2021). External Scientific Report on the interpretation of data from the developmental neurotoxicity *in vitro* testing assays for use in integrated approaches for testing and assessment. EFSA Support. Publ. 18 (10). 10.2903/sp.efsa.2021.en-6924

[B31] Crumpton T. Atkins D. S. Zawia N. H. Barone S. Jr (2001). Lead exposure in pheochromocytoma (PC12) cells alters neural differentiation and Sp1 DNA-binding. Neurotoxicology 22 (1), 49–62. 10.1016/s0161-813x(00)00008-5 11307851

[B32] Culbreth M. Nyffeler J. Willis C. Harrill J. A. (2022). Optimization of human neural progenitor cells for an imaging-based high-throughput phenotypic profiling assay for developmental neurotoxicity screening. Front. Toxicol. 3, 803987. 10.3389/ftox.2021.803987 35295155 PMC8915842

[B33] Cushing M. C. Anseth K. S. (2007). Materials science. Hydrogel cell cultures. Sci. (New York, N.Y.) 316 (5828), 1133–1134. 10.1126/science.1140171 17525324

[B34] D’Avanzo C. Aronson J. Kim Y. H. Choi S. H. Tanzi R. E. Kim D. Y. (2015). Alzheimer's in 3D culture: challenges and perspectives. BioEssays news Rev. Mol. Cell. Dev. Biol. 37 (10), 1139–1148. 10.1002/bies.201500063 PMC4674791 26252541

[B35] del Sol A. Jung S. (2021). The importance of computational modeling in Stem Cell Research. Trends Biotechnol. 39 (2), 126–136. 10.1016/j.tibtech.2020.07.006 32800604

[B36] Dreiman G. H. S. Bictash M. Fish P. V. Griffin L. Svensson F. (2021). Changing the HTS paradigm: AI-driven iterative screening for hit finding. SLAS Discov. Adv. life Sci. R & D 26 (2), 257–262. 10.1177/2472555220949495 PMC7838329 32808550

[B37] Engler A. J. Sen S. Sweeney H. L. Discher D. E. (2006). Matrix elasticity directs stem cell lineage specification. Cell 126 (4), 677–689. 10.1016/j.cell.2006.06.044 16923388

[B38] Engstrom A. Wang H. Xia Z. (2015). Lead decreases cell survival, proliferation, and neuronal differentiation of primary cultured adult neural precursor cells through activation of the JNK and p38 MAP kinases. Toxicol. vitro Int. J. Publ. Assoc. BIBRA 29 (5), 1146–1155. 10.1016/j.tiv.2015.05.001 PMC4457670 25967738

[B39] Gal E. Perin R. Markram H. London M. Segev I. (2020). Neuron geometry underlies universal network features in cortical microcircuits. bioRxiv. 10.1101/656058

[B40] Ghasemi M. Turnbull T. Sebastian S. Kempson I. (2021). The MTT assay: utility, limitations, pitfalls, and interpretation in bulk and single-cell analysis. Int. J. Mol. Sci. 22 (23), 12827. 10.3390/ijms222312827 34884632 PMC8657538

[B41] Giordano G. Costa L. G. (2012). Developmental neurotoxicity: some old and new issues. ISRN Toxicol. 2012, 814795. 10.5402/2012/814795 23724296 PMC3658697

[B42] Grandjean P. Landrigan P. J. (2006). Developmental neurotoxicity of industrial chemicals. Lancet (London, Engl.) 368 (9553), 2167–2178. 10.1016/S0140-6736(06)69665-7 17174709

[B43] Grandjean P. Landrigan P. J. (2014). Neurobehavioural effects of developmental toxicity. Lancet. Neurology 13 (3), 330–338. 10.1016/S1474-4422(13)70278-3 24556010 PMC4418502

[B44] Grienberger C. Konnerth A. (2012). Imaging calcium in neurons. Neuron 73 (5), 862–885. 10.1016/j.neuron.2012.02.011 22405199

[B45] Her G. J. Wu H. C. Chen M. H. Chen M. Y. Chang S. C. Wang T. W. (2013). Control of three-dimensional substrate stiffness to manipulate mesenchymal stem cell fate toward neuronal or glial lineages. Acta biomater. 9 (2), 5170–5180. 10.1016/j.actbio.2012.10.012 23079022

[B46] Hernández-Jerez A. Adriaanse P. Aldrich A. Berny P. Coja T. Duquesne S. (2021). Scientific opinion of the scientific panel on plant protection products and their residues (PPR panel) on testing and interpretation of comparative *in vitro* metabolism studies. EFSA J. 19 (6), e06970. 10.2903/j.efsa.2021.6970 34987623 PMC8696562

[B47] Heydari T. Heidari M. Mashinchian O. Wojcik M. Xu K. Dalby M. J. (2017). Development of a virtual cell model to predict cell response to substrate topography. ACS nano 11 (9), 9084–9092. 10.1021/acsnano.7b03732 28742318

[B48] Heyer D. B. Meredith R. M. (2017). Environmental toxicology: sensitive periods of development and neurodevelopmental disorders. Neurotoxicology 58, 23–41. 10.1016/j.neuro.2016.10.017 27825840

[B49] Hill C. L. Stephens G. J. (2021). An introduction to patch clamp recording. Methods Mol. Biol. Clift. N.J. 2188, 1–19. 10.1007/978-1-0716-0818-0_1 33119844

[B50] Hobson B. D. Choi Se J. Mosharov E. V. Soni R. K. Sulzer D. Sims P. A. (2022). Subcellular proteomics of dopamine neurons in the mouse brain. brain eLife 11, e70921. 10.7554/eLife.70921 35098924 PMC8860448

[B51] Hopkins A. M. DeSimone E. Chwalek K. Kaplan D. L. (2015). 3D *in vitro* modeling of the central nervous system. Prog. Neurobiol. 125, 1–25. 10.1016/j.pneurobio.2014.11.003 25461688 PMC4324093

[B52] Huang F. Shen Q. Zhao J. (2013). Growth and differentiation of neural stem cells in a three-dimensional collagen gel scaffold. Neural Regen. Res. 8 (4), 313–319. 10.3969/j.issn.1673-5374.2013.04.003 25206671 PMC4107534

[B53] Huang G. Wang L. Wang S. Q. Han Y. Wu J. Zhang Q. (2012). Engineering three-dimensional cell mechanical microenvironment with hydrogels. Biofabrication 4 (4), 042001. 10.1088/1758-5082/4/4/042001 23164720

[B54] Huang L. Wang G. (2017). The effects of different factors on the behavior of neural stem cells. Stem cells Int. 2017, 9497325. 10.1155/2017/9497325 29358957 PMC5735681

[B55] Huang M. Li Y. Wu K. Hao S. Cai Q. Zhou Z. J. (2019). Effects of environmental chemicals on the proliferation and differentiation of neural stem cells. Environ. Toxicol. 34 (12), 1285–1291. 10.1002/tox.22829 31400064

[B56] Jiang X. F. Yang K. Yang X. Q. Liu Y. F. Cheng Y. C. Chen X. Y. (2015). Elastic modulus affects the growth and differentiation of neural stem cells. Neural Regen. Res. 10 (9), 1523–1527. 10.4103/1673-5374.165527 26604916 PMC4625521

[B57] Kaja S. Payne A. J. Naumchuk Y. Koulen P. (2017). Quantification of lactate dehydrogenase for cell viability testing using cell lines and primary cultured astrocytes. Curr. Protoc. Toxicol. 72 (2.26), 1–2. 10.1002/cptx.21 PMC5501254 28463416

[B58] Kamata S. Hashiyama R. Hana-Ika H. Ohkubo I. Saito R. Honda A. (2020). Cytotoxicity comparison of 35 developmental neurotoxicants in human induced pluripotent stem cells (iPSC), iPSC-derived neural progenitor cells, and transformed cell lines. Toxicol. vitro Int. J. Publ. Assoc. BIBRA 69, 104999. 10.1016/j.tiv.2020.104999 32949729

[B59] Kang S. Chen X. Gong S. Yu P. Yau S. Su Z. (2017). Characteristic analyses of a neural differentiation model from iPSC-derived neuron according to morphology, physiology, and global gene expression pattern. Sci. Rep. 7 (1), 12233. 10.1038/s41598-017-12452-x 28947763 PMC5612987

[B60] Kang S. Y. Joshi P. Lee M. Y. (2021). High-throughput screening of compound neurotoxicity using 3D-cultured neural stem cells on a 384-pillar plate. Curr. Protoc. 1 (4), e107. 10.1002/cpz1.107 33887124 PMC8075006

[B61] Kapucu F. E. Vinogradov A. Hyvärinen T. Ylä-Outinen L. Narkilahti S. (2022b). Comparative Microelectrode Array Data of the functional development of HPSC-derived and rat neuronal networks. Sci. Data 9 (1), 120. 10.1038/s41597-022-01242-4 35354837 PMC8969177

[B62] Kermani S. Karbalaie K. Madani S. H. Jahangirnejad A. A. Eslaminejad M. B. Nasr-Esfahani M. H. (2008). Effect of lead on proliferation and neural differentiation of mouse bone marrow-mesenchymal stem cells. Toxicol. vitro Int. J. Publ. Assoc. BIBRA 22 (4), 995–1001. 10.1016/j.tiv.2008.02.009 18381235

[B63] Knudsen T. Baker N. Spencer R. Heinonen T. Ellis-Hutchings R. Vargesson N. (2023). “Disruption of VEGFR signaling leading to developmental defects,” in OECD series on adverse outcome pathways. Paris: OECD Publishing. 10.1787/c1116a35-en

[B64] Knudsen T. B. Spencer R. M. Pierro J. D. Baker N. C. (2020). Computational biology and *in silico* toxicodynamics. Curr. Opin. Toxicol. 23-24, 119–126. 10.1016/j.cotox.2020.11.001 36561131 PMC9770085

[B65] Lam D. Enright H. A. Peters S. K. G. Moya M. L. Soscia D. A. Cadena J. (2020). Optimizing cell encapsulation condition in ECM-collagen I hydrogels to support 3D neuronal cultures. J. Neurosci. Methods 329, 108460. 10.1016/j.jneumeth.2019.108460 31626846

[B66] Lan Y. Huang N. Fu Y. Liu K. Zhang H. Li Y. (2022). Morphology-based deep learning approach for predicting osteogenic differentiation. Front. Bioeng. Biotechnol. 9, 802794. 10.3389/fbioe.2021.802794 35155409 PMC8830423

[B67] Langhans S. A. (2018). Three-dimensional *in vitro* cell culture models in drug discovery and drug repositioning. Front. Pharmacol. 9, 6. 10.3389/fphar.2018.00006 29410625 PMC5787088

[B68] Lauvås A. J. Lislien M. Holme J. A. Dirven H. Paulsen R. E. Alm I. M. (2022). Developmental neurotoxicity of acrylamide and its metabolite glycidamide in a human mixed culture of neurons and astrocytes undergoing differentiation in concentrations relevant for human exposure. Neurotoxicology 92, 33–48. 10.1016/j.neuro.2022.07.001 35835329

[B69] Leardini-Tristão M. Andrade G. Garcia C. Reis P. A. Lourenço M. Moreira E. T. S. (2020). Physical exercise promotes astrocyte coverage of microvessels in a model of chronic cerebral hypoperfusion. J. neuroinflammation 17 (1), 117. 10.1186/s12974-020-01771-y 32299450 PMC7161182

[B70] Lee N. H. Cho A. Park S. R. Lee J. W. Sung Taek P. Park C. H. (2018). SERPINB2 is a novel indicator of stem cell toxicity. Cell death & Dis. 9 (7), 724. 10.1038/s41419-018-0748-x PMC6010432 29925837

[B71] Leipzig N. D. Shoichet M. S. (2009). The effect of substrate stiffness on adult neural stem cell behavior. Biomaterials 30 (36), 6867–6878. 10.1016/j.biomaterials.2009.09.002 19775749

[B72] Li Y. Nowak C. M. Pham U. Nguyen K. Bleris L. (2021). Cell morphology-based machine learning models for human cell state classification. NPJ Syst. Biol. Appl. 7 (1), 23. 10.1038/s41540-021-00180-y 34039992 PMC8155075

[B73] Liang Y. Li S. Li Y. Li M. Sun X. An J. (2021). Impact of hydrogel stiffness on the induced neural stem cells modulation. Ann. Transl. Med. 9 (24), 1784. 10.21037/atm-21-6189 35071478 PMC8756230

[B74] Lin S. Schorpp K. Rothenaigner I. Hadian K. (2020). Image-based high-content screening in drug discovery. Drug Discov. today 25 (8), 1348–1361. 10.1016/j.drudis.2020.06.001 32561299

[B75] Lu H. R. Seo M. Kreir M. Tanaka T. Yamoto R. Altrocchi C. (2023). High-throughput screening assay for detecting drug-induced changes in synchronized neuronal oscillations and potential seizure risk based on Ca2+ fluorescence measurements in human induced pluripotent stem cell (hiPSC)-Derived neuronal 2D and 3D cultures. Cells 12 (6), 958. 10.3390/cells12060958 36980298 PMC10046961

[B76] Lu J. Chen M. Qin Y. (2021). Drug-induced cell viability prediction from LINCS-L1000 through WRFEN-XGBoost algorithm. BMC Bioinforma. 22 (1), 13. 10.1186/s12859-020-03949-w PMC7788947 33407085

[B77] Lv D. Hu Z. Lu L. Lu H. Xu X. (2017). Three-dimensional cell culture: a powerful tool in tumor research and drug discovery. Oncol. Lett. 14 (6), 6999–7010. 10.3892/ol.2017.7134 29344128 PMC5754907

[B78] Mai M. Luo S. Fasciano S. Oluwole T. E. Ortiz J. Pang Y. (2023). Morphology-based deep learning approach for predicting adipogenic and osteogenic differentiation of human mesenchymal stem cells (hmscs). Front. Cell Dev. Biol. 11, 1329840. 10.3389/fcell.2023.1329840 38099293 PMC10720363

[B79] Makris S. L. Raffaele K. Allen S. Bowers W. J. Hass U. Alleva E. (2009). A retrospective performance assessment of the developmental neurotoxicity study in support of OECD TEST guideline 426. Environ. Health Perspect. 117 (1), 17–25. 10.1289/ehp.11447 19165382 PMC2627860

[B80] Malik A. R. Willnow T. E. (2019). Excitatory amino acid transporters in physiology and disorders of the central nervous system. Int. J. Mol. Sci. 20 (22), 5671. 10.3390/ijms20225671 31726793 PMC6888459

[B81] Mansel C. Fross S. Rose J. Dema E. Mann A. Hart H. (2019). Lead exposure reduces survival, neuronal determination, and differentiation of P19 stem cells. Neurotoxicology Teratol. 72, 58–70. 10.1016/j.ntt.2019.01.005 30776472

[B82] Mao B. H. Luo Y. K. Wang B. J. Chen C. W. Cheng F. Y. Lee Y. H. (2022). Use of an *in silico* knowledge discovery approach to determine mechanistic studies of silver nanoparticles-induced toxicity from *in vitro* to *in vivo* . Part. fibre Toxicol. 19 (1), 6. 10.1186/s12989-022-00447-0 35031062 PMC8759195

[B83] Margiotta J. F. Smith-Edwards K. M. Nestor-Kalinoski A. Davis B. M. Albers K. M. Howard M. J. (2021). Synaptic components, function and modulation characterized by GCaMP6f Ca2+ imaging in mouse cholinergic myenteric ganglion neurons. Front. physiology 12, 652714. 10.3389/fphys.2021.652714 PMC8365335 34408655

[B84] Marín-Padilla M. (2012). The human brain intracerebral microvascular system: development and structure. Front. Neuroanat. 6, 38. 10.3389/fnana.2012.00038 22993505 PMC3440694

[B85] Masjosthusmann S. Blum J. Bartmann K. Dolde X. Holzer A. Stürzl L. (2020). Establishment of an *a priori* protocol for the implementation and interpretation of an in‐vitro testing battery for the assessment of developmental neurotoxicity. EFSA Support. Publ. 17 (10). 10.2903/sp.efsa.2020.en-1938

[B86] Mattiassi S. Conner A. A. Feng F. Goh E. L. K. Yim E. K. F. (2023). The combined effects of topography and stiffness on neuronal differentiation and maturation using a hydrogel platform. Cells 12 (6), 934. 10.3390/cells12060934 36980275 PMC10047827

[B87] McCready F. P. Gordillo-Sampedro S. Pradeepan K. Martinez-Trujillo J. Ellis J. (2022). Multielectrode arrays for functional phenotyping of neurons from induced pluripotent stem cell models of neurodevelopmental disorders. Biology 11 (2), 316. 10.3390/biology11020316 35205182 PMC8868577

[B88] Mitchell K. Iadarola M. J. (2010). RT-PCR analysis of pain genes: use of gel-based RT-PCR for studying induced and tissue-enriched gene expression. Methods Mol. Biol. Clift. N.J. 617, 279–295. 10.1007/978-1-60327-323-7_21 PMC3417750 20336429

[B89] Mito T. Konomi H. Houdou S. Takashima S. (1991). Immunohistochemical study of the vasculature in the developing brain. Pediatr. Neurol. 7 (1), 18–22. 10.1016/0887-8994(91)90100-y 2029288

[B90] Morrison S. J. (2001). Neuronal potential and lineage determination by neural stem cells. Curr. Opin. Cell Biol. 13 (6), 666–672. 10.1016/s0955-0674(00)00269-6 11698181

[B91] National Research Council US. Committee on neurotoxicology and models for assessing risk. Environmental neurotoxicology. Washington (DC): National Academies Press US. Available at: https://www.ncbi.nlm.nih.gov/books/NBK234243.

[B92] Obergrussberger A. Rinke-Weiß I. Goetze T. A. Rapedius M. Brinkwirth N. Becker N. (2022). The suitability of high throughput automated patch clamp for physiological applications. J. physiology 600 (2), 277–297. 10.1113/JP282107 34555195

[B93] OECD (2007). Test No. 426: developmental neurotoxicity study, OECD guidelines for the testing of chemicals, section 4. Paris: OECD Publishing. 10.1787/9789264067394-en

[B94] OECD (2008). OECD series on testing and assessment, number 43: guidance document on mammalian reproductive toxicity testing and assessment. Paris, ENV/JM/MONO: OECD Publishing, 16.

[B95] OECD (2017). OECD series on testing and assessment, number 261: report of the oecd/EFSA workshop on developmental neurotoxicity (DNT): the use OF non-animal test methods for regulatory purposes. Paris: OECD Publishing. ENV/JM/MONO(2017)4. 10.14573/altex.1701171 28407175

[B96] OECD (2018). Test No. 443: extended one-generation reproductive toxicity study, OECD guidelines for the testing of chemicals, section 4. Paris: OECD Publishing. 10.1787/9789264185371-en

[B97] OECD (2022). Draft of initial guidance document on evaluation of data from the developmental neurotoxicity (DNT) in-vitro testing battery. Paris: OECD Publishing. Available at: https://www.oecd.org/chemicalsafety/testing/guidance-evaluation-of-data-developmental-neurotoxicity-in-vitro-testing.pdf.

[B98] OECD (2023). OECD series on testing and assessment, number 377: initial recommendations on evaluation of data from the developmental neurotoxicity (DNT) in-vitro testing battery. Paris, ENV/CBC/MONO: OECD Publishing. 10.1787/91964ef3-en

[B99] Organisation for EconomicCo-operation and Development (OECD). *In vitro* assays for developmental neurotoxicity. Organisation for Economic Co-operation and Development (OECD). (n.d.). Available at: https://www.oecd.org/en/topics/sub-issues/testing-of-chemicals/in-vitro-assays-for-developmental-neurotoxicity.html .

[B100] Okano H. Temple S. (2009). Cell types to order: temporal specification of CNS stem cells. Curr. Opin. Neurobiol. 19 (2), 112–119. 10.1016/j.conb.2009.04.003 19427192

[B101] Ortinau S. Schmich J. Block S. Liedmann A. Jonas L. Weiss D. G. (2010). Effect of 3D-scaffold formation on differentiation and survival in human neural progenitor cells. Biomed. Eng. online 9, 70. 10.1186/1475-925X-9-70 21070668 PMC2996398

[B102] Pardridge W. M. (1983). Brain metabolism: a perspective from the blood-brain barrier. Physiol. Rev. 63 (4), 1481–1535. 10.1152/physrev.1983.63.4.1481 6361813

[B103] Park S. Nguyen T. Benoit E. Sackett D. L. Garmendia-Cedillos M. Pursley R. (2021). Quantitative evaluation of the dynamic activity of HeLa cells in different viability states using dynamic full-field optical coherence microscopy. Biomed. Opt. express 12 (10), 6431–6441. 10.1364/BOE.436330 34745747 PMC8548024

[B104] Park S. Veluvolu V. Martin W. S. Nguyen T. Park J. Sackett D. L. (2022). Label-free, non-invasive, and repeatable cell viability bioassay using dynamic full-field optical coherence microscopy and supervised machine learning. Biomed. Opt. express 13 (6), 3187–3194. 10.1364/BOE.452471 35781969 PMC9208588

[B105] Passaro A. P. Stice S. L. (2021). Electrophysiological analysis of brain organoids: current approaches and advancements. Front. Neurosci. 14, 622137. 10.3389/fnins.2020.622137 33510616 PMC7835643

[B106] Pei Y. Peng J. Behl M. Sipes N. S. Shockley K. R. Rao M. S. (2016). Comparative neurotoxicity screening in human iPSC-derived neural stem cells, neurons and astrocytes. Brain Res. 1638, 57–73. 10.1016/j.brainres.2015.07.048 26254731 PMC5032144

[B107] Pek Y. S. Wan A. C. Ying J. Y. (2010). The effect of matrix stiffness on mesenchymal stem cell differentiation in a 3D thixotropic gel. Biomaterials 31 (3), 385–391. 10.1016/j.biomaterials.2009.09.057 19811817

[B108] Pistollato F. de Gyves E. M. Carpi D. Bopp S. K. Nunes C. Worth A. (2020). Assessment of developmental neurotoxicity induced by chemical mixtures using an adverse outcome pathway concept. Environ. health a Glob. access Sci. source 19 (1), 23. 10.1186/s12940-020-00578-x PMC7038628 32093744

[B109] Pontes Soares C. Midlej V. de Oliveira M. E. Benchimol M. Costa M. L. Mermelstein C. (2012). 2D and 3D-organized cardiac cells shows differences in cellular morphology, adhesion junctions, presence of myofibrils and protein expression. PloS one 7 (5), e38147. 10.1371/journal.pone.0038147 22662278 PMC3360656

[B110] Porciúncula L. O. Goto-Silva L. Ledur P. F. Rehen S. K. (2021). The age of brain organoids: tailoring cell identity and functionality for normal brain development and disease modeling. Front. Neurosci. 15, 674563. 10.3389/fnins.2021.674563 34483818 PMC8414411

[B111] Rammensee S. Kang M. S. Georgiou K. Kumar S. Schaffer D. V. (2017). Dynamics of mechanosensitive neural stem cell differentiation. Stem cells Dayt. Ohio 35 (2), 497–506. 10.1002/stem.2489 PMC5285406 27573749

[B112] Renner H. Grabos M. Becker K. J. Kagermeier T. E. Wu J. Otto M. (2020). A fully automated high-throughput workflow for 3D-based chemical screening in human midbrain organoids. eLife 9, e52904. 10.7554/eLife.52904 33138918 PMC7609049

[B113] Rice D. Barone S.(2000). Critical periods of vulnerability for the developing nervous system: evidence from humans and animal models. Environ. health Perspect. 108 (Suppl. 3), 511–533. 10.1289/ehp.00108s3511 10852851 PMC1637807

[B114] Riss T. Niles A. Moravec R. Karassina N. Vidugiriene J. (2019). “Cytotoxicity assays: *in vitro* methods to measure dead cells,” in Assay guidance manual. Editor Markossian S., (Bethesda, MD: Eli Lilly & Company and the National Center for Advancing Translational Sciences). 31070879

[B115] Sagar Grün D. (2020). Deciphering cell fate decision by integrated single-cell sequencing analysis. Annu. Rev. Biomed. data Sci. 3, 1–22. 10.1146/annurev-biodatasci-111419-091750 32780577 PMC7115822

[B116] Saili K. S. Zurlinden T. J. Schwab A. J. Silvin A. Baker N. C. Hunter E. S. 3rd (2017). Blood-brain barrier development: systems modeling and predictive toxicology. Birth defects Res. 109 (20), 1680–1710. 10.1002/bdr2.1180 29251840 PMC6476421

[B117] Schaudien D. Harleman J. H. Bouallala F. Kuper C. F. (2018). Lymphoid tissue and pathological influences of toxicants. Compr. Toxicol., 322–342. 10.1016/b978-0-08-100601-6.01990-6

[B118] Seal S. Carreras-Puigvert J. Trapotsi M. A. Yang H. Spjuth O. Bender A. (2022). Integrating cell morphology with gene expression and chemical structure to aid mitochondrial toxicity detection. Commun. Biol. 5, 858. 10.1038/s42003-022-03763-5 35999457 PMC9399120

[B119] Seal S. Yang H. Vollmers L. Bender A. (2021). Comparison of cellular morphological descriptors and molecular fingerprints for the prediction of cytotoxicity- and proliferation-related assays. Chem. Res. Toxicol. 34 (2), 422–437. 10.1021/acs.chemrestox.0c00303 33522793

[B120] Seo J. Shin J. Y. Leijten J. Jeon O. Camci-Unal G. Dikina A. D. (2018). High-throughput approaches for screening and analysis of cell behaviors. Biomaterials 153, 85–101. 10.1016/j.biomaterials.2017.06.022 29079207 PMC5702937

[B121] Shafer T. J. Brown J. P. Lynch B. Davila-Montero S. Wallace K. Friedman K. P. (2019). Evaluation of chemical effects on network formation in cortical neurons grown on microelectrode arrays. Toxicol. Sci. official J. Soc. Toxicol. 169 (2), 436–455. 10.1093/toxsci/kfz052 30816951

[B122] Singh A. P. Zheng X. Lin-Schmidt X. Chen W. Carpenter T. J. Zong A. (2020). Development of a quantitative relationship between CAR-affinity, antigen abundance, tumor cell depletion and CAR-T cell expansion using a multiscale systems PK-PD model. mAbs 12 (1), 1688616. 10.1080/19420862.2019.1688616 31852337 PMC6927769

[B123] Sisnaiske J. Hausherr V. Krug A. K. Zimmer B. Hengstler J. G. Leist M. (2014). Acrylamide alters neurotransmitter induced calcium responses in murine ESC-derived and primary neurons. Neurotoxicology 43, 117–126. 10.1016/j.neuro.2014.03.010 24726791

[B124] Soltani M. H. Pichardo R. Song Z. Sangha N. Camacho F. Satyamoorthy K. (2005). Microtubule-associated protein 2, a marker of neuronal differentiation, induces mitotic defects, inhibits growth of melanoma cells, and predicts metastatic potential of cutaneous melanoma. Am. J. pathology 166 (6), 1841–1850. 10.1016/S0002-9440(10)62493-5 PMC1602405 15920168

[B125] Song C. Kanthasamy A. Kanthasamy A. (2011). Cell signaling mechanisms in developmental neurotoxicity. Reproductive Dev. Toxicol., 835–845. 10.1016/b978-0-12-382032-7.10063-3

[B126] Strober W. (2015). Trypan blue exclusion test of cell viability. Curr. Protoc. Immunol. 111, A3.B.1–A3.B.3. 10.1002/0471142735.ima03bs111 PMC6716531 26529666

[B127] Stukel J. M. Willits R. K. (2018). The interplay of peptide affinity and scaffold stiffness on neuronal differentiation of neural stem cells. Biomed. Mater. Bristol, Engl. 13 (2), 024102. 10.1088/1748-605X/aa9a4b 29133625

[B128] Tibbitt M. W. Anseth K. S. (2009). Hydrogels as extracellular matrix mimics for 3D cell culture. Biotechnol. Bioeng. 103 (4), 655–663. 10.1002/bit.22361 19472329 PMC2997742

[B129] Toh M. F. Brooks J. M. Strassmaier T. Haedo R. J. Puryear C. B. Roth B. L. (2020). Application of high-throughput automated patch-clamp electrophysiology to study voltage-gated ion channel function in primary cortical cultures. SLAS Discov. Adv. life Sci. R & D 25 (5), 447–457. 10.1177/2472555220902388 32003306

[B130] TSCA chemical substance inventory. (2023). Available at: https://www.epa.gov/tsca-inventory

[B131] Udvary D. Harth P. Macke J. H. Hege H. C. de Kock C. P. J. Sakmann B. (2022). The impact of neuron morphology on cortical network architecture. Cell Rep. 39 (2), 110677. 10.1016/j.celrep.2022.110677 35417720 PMC9035680

[B132] U.S. Department of Health and Human Services (2023). Adverse outcome pathways. Natl. Inst. Environ. Health Sci. Available at: https://ntp.niehs.nih.gov/whatwestudy/niceatm/comptox/ct-aop/aop.

[B133] USEPA (2021). New approach methods work plan (v2). Washington, DC: U.S. Environmental Protection Agency. EPA/600/X-21/209.

[B134] USEPA (United States Environmental Protection Agency) (1998). Developmental neurotoxicity study. Washington, DC, USA: USEPA. Health effects test guidelines. OPPTS 870.6300.

[B135] Valdiglesias V. Kiliç G. Costa C. Fernández-Bertólez N. Pásaro E. Teixeira J. P. (2015). Effects of iron oxide nanoparticles: cytotoxicity, genotoxicity, developmental toxicity, and neurotoxicity. Environ. Mol. Mutagen. 56 (2), 125–148. 10.1002/em.21909 25209650

[B136] Vallejo-Giraldo C. Genta M. Cauvi O. Goding J. Green R. (2020). Hydrogels for 3D neural tissue models: understanding cell-material interactions at a molecular level. Front. Bioeng. Biotechnol. 8, 601704. 10.3389/fbioe.2020.601704 33240868 PMC7677185

[B137] van der Graaf P. (2018). Faculty opinions recommendation of mechanistic models versus machine learning, a fight worth fighting for the biological community? Fac. Opin. – Post-Publication Peer Rev. Biomed. Literature. 10.3410/f.733245614.793546034

[B138] Viswanathan S. Zandstra P. W. (2003). Towards predictive models of stem cell fate. Cytotechnology 41 (2-3), 75–92. 10.1023/A:1024866504538 19002945 PMC3466685

[B139] Wang J. Chen Y. Chen B. Ding J. Xia G. Gao C. (2010). Pharmacokinetic parameters and tissue distribution of magnetic Fe(3)O(4) nanoparticles in mice. Int. J. nanomedicine 5, 861–866. 10.2147/IJN.S13662 21042548 PMC2963932

[B140] Wang Y. Jeon H. (2022). 3D cell cultures toward quantitative high-throughput drug screening. Trends Pharmacol. Sci. 43 (7), 569–581. 10.1016/j.tips.2022.03.014 35504760

[B141] Way G. P. Kost-Alimova M. Shibue T. Harrington W. F. Gill S. Piccioni F. (2021). Predicting cell health phenotypes using image-based morphology profiling. Mol. Biol. cell 32 (9), 995–1005. 10.1091/mbc.E20-12-0784 33534641 PMC8108524

[B142] Woodruff G. Phillips N. Carromeu C. Guicherit O. White A. Johnson M. (2020). Screening for modulators of neural network activity in 3D human iPSC-derived cortical spheroids. PloS one 15 (10), e0240991. 10.1371/journal.pone.0240991 33091047 PMC7581002

[B143] Wu J. Ding T. Sun J. (2013). Neurotoxic potential of iron oxide nanoparticles in the rat brain striatum and hippocampus. Neurotoxicology 34, 243–253. 10.1016/j.neuro.2012.09.006 22995439

[B144] Xu T. Molnar P. Gregory C. Das M. Boland T. Hickman J. J. (2009). Electrophysiological characterization of embryonic hippocampal neurons cultured in a 3D collagen hydrogel. Biomaterials 30 (26), 4377–4383. 10.1016/j.biomaterials.2009.04.047 19501396

[B145] Zablotsky B. Black L. I. Maenner M. J. Schieve L. A. Danielson M. L. Bitsko R. H. (2019). Prevalence and trends of developmental disabilities among children in the United States: 2009-2017. Pediatrics 144 (4), e20190811. 10.1542/peds.2019-0811 31558576 PMC7076808

[B146] Zare-Mehrjardi N. Khorasani M. T. Hemmesi K. Mirzadeh H. Azizi H. Sadatnia B. (2011). Differentiation of embryonic stem cells into neural cells on 3D poly (D, L-lactic acid) scaffolds versus 2D cultures. Int. J. Artif. organs 34 (10), 1012–1023. 10.5301/ijao.5000002 22161284

[B147] Zhang J. Jiao J. (2015). Molecular biomarkers for embryonic and adult neural stem cell and neurogenesis. BioMed Res. Int. 2015, 727542. 10.1155/2015/727542 26421301 PMC4569757

[B148] Zhang Y. Rózsa M. Liang Y. Bushey D. Wei Z. Zheng J. (2023). Fast and sensitive GCaMP calcium indicators for imaging neural populations. Nature 615 (7954), 884–891. 10.1038/s41586-023-05828-9 36922596 PMC10060165

[B149] Zhang Z. Chen L. Humphries B. Brien R. Wicha M. S. Luker K. E. (2018). Morphology-based prediction of cancer cell migration using an artificial neural network and a random decision forest. Integr. Biol. quantitative Biosci. nano macro 10 (12), 758–767. 10.1039/c8ib00106e PMC6329292 30420987

[B150] Zhao Q. Dai W. Chen H. Y. Jacobs R. E. Zlokovic B. V. Lund B. T. (2022). Prenatal disruption of blood-brain barrier formation via cyclooxygenase activation leads to lifelong brain inflammation. Proc. Natl. Acad. Sci. United States of America 119 (15), e2113310119. 10.1073/pnas.2113310119 PMC9169666 35377817

[B151] Zhao X. Moore D. L. (2018). Neural stem cells: developmental mechanisms and disease modeling. Cell tissue Res. 371 (1), 1–6. 10.1007/s00441-017-2738-1 29196810 PMC5963504

[B152] Zhou Z. Zhu J. Jiang M. Sang L. Hao K. He H. (2021). The combination of cell cultured technology and *in silico* model to inform the drug development. Pharmaceutics 13 (5), 704. 10.3390/pharmaceutics13050704 34065907 PMC8151315

[B153] Zhu T. Cao S. Su P. C. Patel R. Shah D. Chokshi H. B. (2013). Hit identification and optimization in virtual screening: practical recommendations based on a critical literature analysis. J. Med. Chem. 56 (17), 6560–6572. 10.1021/jm301916b 23688234 PMC3772997

[B154] Zhu Y. Huang R. Wu Z. Song S. Cheng L. Zhu R. (2021). Deep learning-based predictive identification of neural stem cell differentiation. Nat. Commun. 12 (1), 2614. 10.1038/s41467-021-22758-0 33972525 PMC8110743

[B155] Zurlinden T. J. Saili K. S. Rush N. Kothiya P. Judson R. S. Houck K. A. (2020). Profiling the ToxCast library with a pluripotent human (H9) stem cell line-based biomarker assay for developmental toxicity. Toxicol. Sci. official J. Soc. Toxicol. 174 (2), 189–209. 10.1093/toxsci/kfaa014 PMC8527599 32073639

